# Lineage, Antimicrobial Resistance and Virulence of *Citrobacter* spp

**DOI:** 10.3390/pathogens9030195

**Published:** 2020-03-06

**Authors:** Liyun Liu, Liyun Qin, Shuai Hao, Ruiting Lan, Baohong Xu, Yumei Guo, Ruiping Jiang, Hui Sun, Xiaoping Chen, Xinchao LV, Jianguo Xu, Chuan Zhao

**Affiliations:** 1 State Key Laboratory of Infectious Disease Prevention and Control, National Institute for Communicable Disease Control and Prevention, Chinese Center for Disease Control and Prevention, Beijing 102206, China; sunhui@icdc.cn (H.S.); chenxiaoping@icdc.cn (X.C.); xujianguo@icdc.cn (J.X.); 2 Shijiazhuang Center for Disease Control and Prevention, Shijiazhuang 050011, China; qinliyun-2007@163.com (L.Q.); Wsws1120@163.com (B.X.); Guokexin2199@163.com (Y.G.); Jrpok97@163.com (R.J.); lvxinzhao1986@163.com (X.L.); 3 Beijing Advanced Innovation Center for Food Nutrition and Human Health, Beijing Engineering and Technology Research Center of Food Additives, Beijing Technology and Business University, Beijing 100048, China; haoshuai@btbu.edu.cn; 4 School of Biotechnology and Biomolecular Sciences, University of New South Wales, Sydney, NSW 2052, Australia; r.lan@unsw.edu.au

**Keywords:** *Citrobacter*, sequence types, multidrug resistance, adhesion, cytotoxicity

## Abstract

*Citrobacter* spp. are opportunistic human pathogens which can cause nosocomial infections, sporadic infections and outbreaks. In order to determine the genetic diversity, *in vitro* virulence properties and antimicrobial resistance profiles of *Citrobacter* spp., 128 *Citrobacter* isolates obtained from human diarrheal patients, foods and environment were assessed by multilocus sequence typing (MLST), antimicrobial susceptibility testing and adhesion and cytotoxicity testing to HEp-2 cells. The 128 *Citrobacter* isolates were typed into 123 sequence types (STs) of which 101 were novel STs, and these STs were divided into five lineages. Lineages I and II contained *C. freundii* isolates; Lineage III contained all *C. braakii* isolates, while Lineage IV and V contained *C. youngae* isolates. Lineages II and V contained more adhesive and cytotoxic isolates than Lineages I, III, and IV. Fifty-one of the 128 isolates were found to be multidrug-resistant (MDR, ≥3) and mainly distributed in Lineages I, II, and III. The prevalence of quinolone resistance varied with Lineage III (*C. braakii*) having the highest proportion of resistant isolates (52.6%), followed by Lineage I (*C. freundii*) with 23.7%. Seven *qnrB* variants, including two new alleles (*qnrB93* and *qnrB94*) were found with Lineage I being the main reservoir. In summary, highly cytotoxic MDR isolates from diarrheal patients may increase the risk of severe disease.

## 1. Background

The genus *Citrobacter* contained 11 species, most of which are opportunistic human pathogens that can cause nosocomial infections [1], and a range of other infections [2,3,4,5,6]. In this study, we focus on three *Citrobacter* species, *C. freundii, C. youngae* and *C. braakii,* as potential foodborne pathogens. *C. freundii* is the most commonly isolated *Citrobacter* species causing diarrhea and other infections [7,8], while *C. youngae* and *C. braakii* are rarely reported to cause infections. Some *C. freundii* strains caused food poisoning or diarrhea in humans which were found to carry virulent factors, such as Shiga-like toxins, heat-stable toxins, or virulence islands [9,10]. *C. youngae* can cause peritonitis [11]. *C. braakii* has been isolated from the peritonea of acute peritonitis patients, as well as from food products [12,13,14,15]. In our previous studies, *C. freundii* and *C. youngae* have been isolated from the fecal samples of diarrheal patients and different types of food samples, and are potential foodborne pathogens [10,16], while *C. braakii* has been isolated from food [10].

Antibiotic resistance of *Citrobacter* has increased, and multidrug-resistant (MDR) isolates have frequently been reported [10,17,18,19,20,21]. Frequent isolation of MDR *C. freundii* with resistance to β-lactams, quinolones and aminoglycosides has been reported by several international surveillance programs [18]. In our previous study, 31.7% of *C. freundii* isolates were MDR that were resistant to β-lactams, quinolones, aminoglycosides, tetracyclines, phenicols, sulfonamides or nitrofuran [10,16]. Furthermore, although not MDR, 4.9% were also resistant to aminoglycosides, β-lactams, and quinolones [10,16].

Antibiotic resistant *Citrobacter* often harbored extended-spectrum β-lactamase (ESBL) [1,19,20], and plasmid-mediated quinolone resistance (PMQR) determinants [21]. The prevalence of ESBL and PMQR *Citrobacter* isolates was reported from several international studies [1,19,20,21]. In our previous study, we identified four *C. freundii* isolates from clinical sources and foods that were ESBL producing and 21 isolates from clinical sources and foods that harbored PMQR genes, including *aac(6’)-Ib-cr*, *qnrS1*, *qnrB9*, *qnrB13*, *qnrB16*, *qnrB17*, *qnrB63*, *qnrB76*, *qnrB77*, or *qnrB92* [10,16].

Fluoroquinolone resistance is associated with mutations in DNA gyrase and topoisomerase IV genes, in particular, mutations in the quinolone resistance-determining regions (QRDRs) of *gyrA* and *parC* genes [22]. It has been reported that *C. freundii* isolates with reduced susceptibility to fluoroquinolones were found to contain Thr83Ile or Asp87Asn mutation in *gyrA* [22,23]. In our previous study, we screened mutations in the QRDRs of *gyrA* and *parC* genes in fluoroquinolone resistant isolates. Four of the six fluoroquinolone resistant isolates were found to carry Thr59Ile, Gln111Arg and Ile134Val mutations of the *gyrA* gene [16]. However, it remains to be determined whether these mutations confer resistance to fluoroquinolones. No mutations in the QRDR region of the *parC* gene was found in the six fluoroquinolones resistant isolates [16].

To further understand the genetic diversity, virulence and antibiotic resistance of *Citrobacter spp*. From different sources, in this study, we isolated 128 *Citrobacter* isolates from diarrheal outpatients, food and environment in Shijiazhuang Hebei Province, China. We performed multilocus sequence typing (MLST) to determine the relationships of the isolates and screened for *bla*_CTX-M_, *bla*_SHV_, *bla*_TEM_ and *qnr* genes by PCR and mutations in *gyrA* and *parC* genes by PCR sequencing and assayed the adhesion and cytotoxicity to Hep-2 cells of all isolates.

## 2. Results 

### 2.1. Multilocus Sequence Typing of Citrobacter Isolates

**Figure 1:**
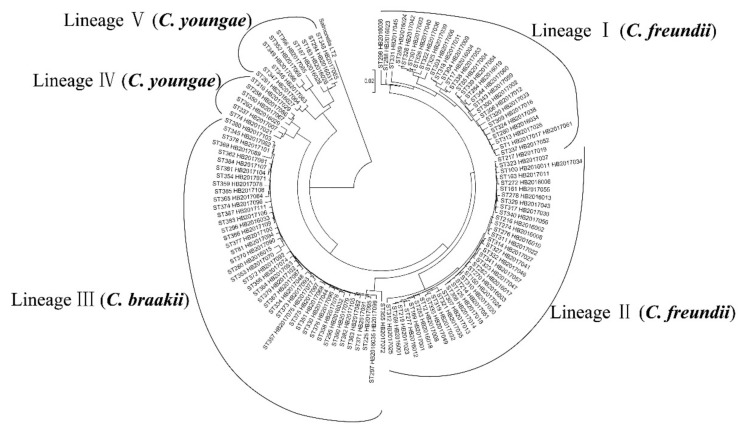
Phylogenetic relationships of the 128 *Citrobacter* isolates from this study. The phylogenetic tree of the 128 *Citrobacter* isolates was constructed using the concatenated sequences of the seven housekeeping genes by the neighbor-joining algorithm. *Salmonella* LT2 was used as an outgroup. Lineage divisions were marked. Bootstrap values of 50% or more from 1000 replicates were shown.

The 128 *Citrobacter* isolates were typed into 123 STs with the 67 *C. freundii* isolates dividing into 65 STs, the 45 *C. braakii* isolates into 42 STs and the 16 *C. younga*e isolates into 16 STs (Table 1 and Figure 1). Of the 123 STs, 101 were novel STs (from ST269 to ST387). No STs were predominant. Two STs (ST1 and ST100) of *C. freundii* each contained two isolates. ST1 contained one isolate from a diarrheal patient and one from food. ST100 contained one isolate from the environment and one from food. ST357 and ST375 of *C. braakii* each contained two isolates from diarrheal patients or foods. ST297 of *C. braakii* contained two isolates, with one from the environment and one from food.

The concatenated sequence of the seven housekeeping genes for the 128 *Citrobacter* isolates, was used to construct a neighbor-joining tree (Figure 1). *Salmonella* LT2 was used as an outgroup. The 128 *Citrobacter* isolates were divided into five lineages with strong bootstrap support to the lineage divisions. Lineages I and II contained *C. freundii* isolates, while Lineages IV and V contained *C. youngae* isolates exclusively. Lineage III contained all *C. braakii* isolates. *Citrobacter* isolates from different sources were not grouped by source and were dispersed among different lineages (Table 1 and Figure 1).

**Figure 2:**
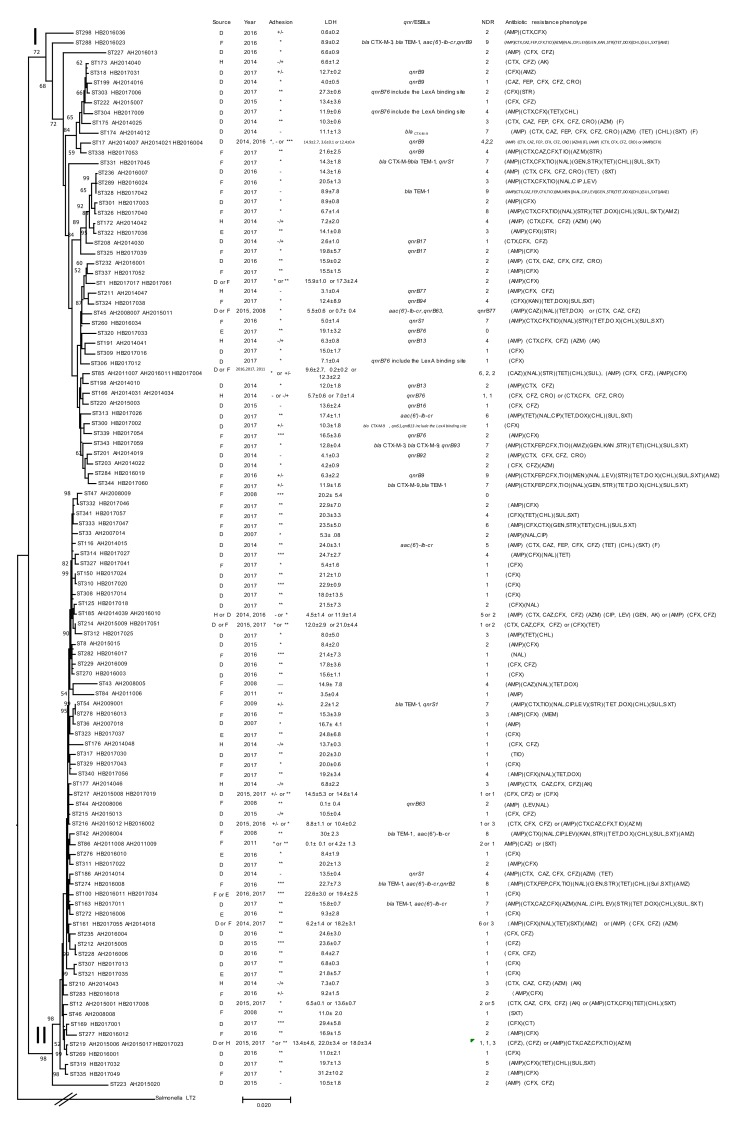
Phylogenetic relationships of the 123 *C. freundii* isolates from this study and two previous studies [10,16]. Lineages are marked on the node with roman numerals. Bootstrap values from 1000 replicates are shown on or near the nodes if ≥50%. The presence of ESBL and *qnr* genes, source, year, NDR (number of drugs resistant to), adhesion, LDH and antibiotic resistance phenotype of an isolate is shown on the right. The tree was constructed using the neighbor joining method. ST, D, F, E, H, and LDH denote sequence types, isolates from diarrheal patients, foods, environment and healthy individuals, and lactate dehydrogenase, respectively. Adhesion index: ***, >50; **, >1 and <50; *, <1; +/-, ambivalent or no adhesion; -, no adhesion.

The STs of *C. freundii* in Lineages I and II from this study were compared with 85 STs of *C. freundii* from Maanshan Anhui Province in our previous study [10,16], only nine STs found in this study shared the same STs from Maanshan city. *C. freundii* from these two regions displayed high diversity. All STs of *C. freundii* from this study and our previous study were used to construct a phylogenetic tree. The tree was divided into six clusters with strong bootstrap support of the cluster divisions (Figure 2 and Supplementary Figure S1). Cluster 1 to 5 were the same as previously defined [16]. However, a group within cluster 1 of our previous study is now a separate cluster and named cluster 6. The STs of *C. freundii* in Lineage I and Lineage II from this study were equivalent to cluster 1 and cluster 2 in our previous study (Figure 2 and Supplementary Figure S1).

**Figure 3:**
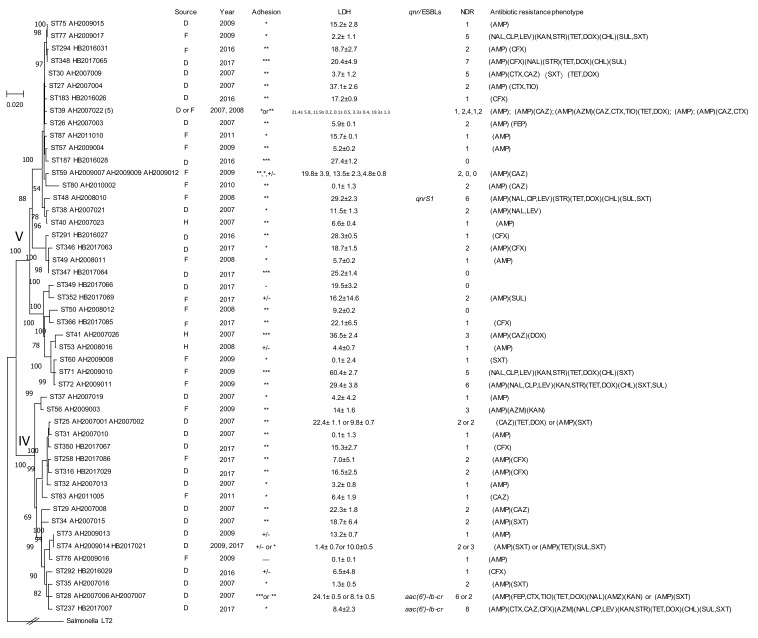
Phylogenetic relationships of the 57 *C. youngae* isolates from this study and our previous study [10]. Lineages divisions are marked on the node with roman numerals. The number in bracket after strain name denote number of strains for ST39 which includes AH2007022, AH2007024, AH2007025, AH2008001, AH2008002). Bootstrap values (numbers on or near the nodes) from 1000 replicates are shown if ≥50%. The presence of ESBLs and *qnr* genes, source, year, NDR (the number of drugs resistant to), adhesion, LDH and antibiotic resistance phenotype of an isolate is shown on the right. The tree was constructed using the neighbor joining method. ST, D, F, H, and LDH denote sequence types, isolates from diarrheal patients, foods and healthy individuals, and lactate dehydrogenase, respectively. Adhesion index: ***, >50; **, >1 and <50; *, <1; +/-, ambivalent or no adhesion; -, no adhesion.

**Figure 4:**
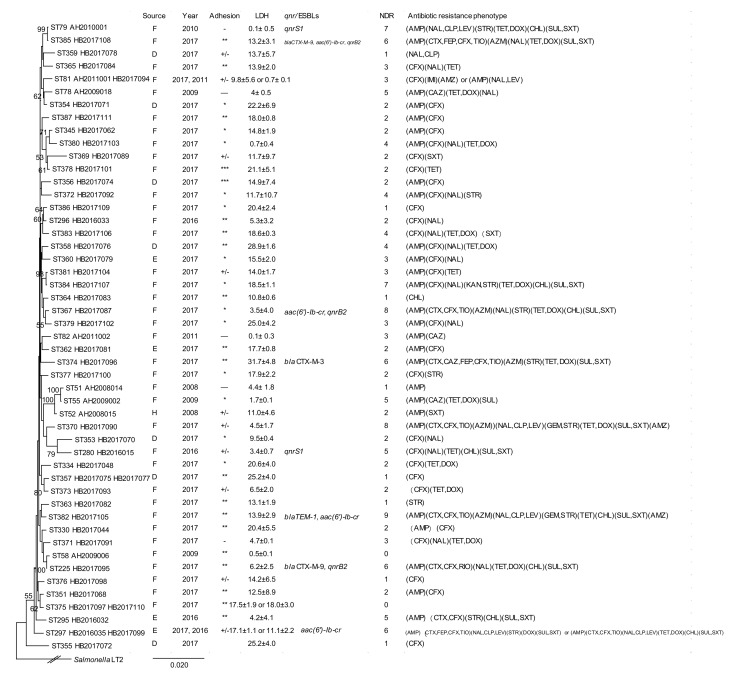
Phylogenetic relationships of the 53 *C. braakii* isolates (lineage III) from this study and our previous study [10]. The phylogenetic tree of the 53 *C. braakii* isolates was constructed using the concatenated sequences of the seven housekeeping genes by the neighbor-joining method. Bootstrap values of 50% or more from 1000 replicates were shown. The presence of ESBLs and *qnr* genes, source, year, NDR (number of drugs resistant to), adhesion, LDH and antibiotic resistance phenotype of an isolate is shown on the right. The tree was constructed using the neighbor joining algorithm. ST, D, F, H, and LDH indicate sequence types, isolates from diarrheal patients, foods and healthy individuals, and lactate dehydrogenase, respectively. Adhesion index: ***, >50; **, >1 and <50; *, <1; +/-, ambivalent or no adhesion; -, no adhesion.

The 16 STs of *C. youngae* in Lineage IV and Lineage V from this study were compared with 32 STs of *C. youngae* from Maanshan, Anhui Province in our previous study. Fifteen STs from this study were novel STs, and there was little overlap of STs. All STs of *C. youngae* from this study and our previous study were used to construct a phylogenetic tree, the tree was divided into two clusters (cluster 1 and cluster 2) with strong bootstrap support of the cluster divisions, and 16 STs of *C. youngae* in Lineage IV and Lineage V from this study were equivalent to cluster 1 and cluster 2 in our previous study [10] (Figure 3),

The 42 STs of *C. braakii* in Lineage III from this study were compared with 8 STs of *C. braakii* from Maanshan Anhui Province in our previous study [10], and only one ST was common between the two regions. All STs of *C. braakii* from this study and our previous study were used to construct a phylogenetic tree (Figure 4), and all isolates belonged to the same cluster.

We further analyzed the 123 STs using eBURST [24,25] to identify clonal complexes. In this study we defined CCs as STs shared six of the seven alleles to identify the most closely related STs [24,25]. We also retrieved all *C.* *freundii* STs from other countries from the public MLST database to identify CCs that include isolates from other countries. It should be noted that there is no *C. youngae* or *C. braakii* isolates from other countries in the MLST database. There were 27, 7 and 5 CCs identified for *C. freundii*, *C. youngae* and *C. braakii* isolates, respectively (Supplementary Table S1). For the 27 *C.* *freundii* CCs, 17 CCs included isolates from other countries with one CC containing isolates from five different countries, 10 CCs included isolates from two different regions of China but no isolates from other countries, and only four CCs were restricted in the same region of China. For the seven *C. youngae* CCs, only one was from two different regions of China. For the five *C. braakii* CCs only one was from two different regions of China also. 

### 2.2. Prevalence of Antimicrobial Resistance

Susceptibility to 22 antibiotics was tested on the 128 *Citrobacter* isolates using the broth microdilution method according to CLSI recommendations (Table 2). All isolates were sensitive to amikacin (AMI). For the 67 *C. freundii* isolates, most were resistant to one or more of the β-lactams, especially to penicillins (58.2%), cephalosporins (9.0–94.0%), monobactams (7.5%) and carbapenems (1.5–4.5%). Resistance to the three quinolones tested ranged from 7.5% to 23.9%. Resistance to other antibiotics was as follows: Aminoglycosides (0–20.9%), tetracyclines (16.4–32.8%), phenicols (25.4%), sulfonamides (22.4–25.4%) and macrolides (10.4%). For the 45 *C. braakii* isolates, resistance to different β-lactams was as follows: Penicillins (51.1%), cephalosporins (3.0–88.9%), monobactams (11.1%) and carbapenems (0–2.2%), and resistance to quinolones (6.7–44.4%), aminoglycosides (0–22.2%), tetracyclines (31.1–42.2%), phenicols (20.0%), sulfonamides (24.4–28.9%) and macrolides (6.7%). For 16 *C. youngae* isolates, varied resistance was found to penicillins (50.0%), cephalosporins (0–68.8%), monobactams (6.3%), quinolones (6.3–12.5%), aminoglycosides (0–12.5%), tetracyclines (12.5–18.8%), phenicols (12.5%) and sulfonamides (18.8%).

Resistance to at least one antibiotic of three or more distinct classes was defined as multidrug-resistant (MDR). We found 51 MDR isolates which were distributed in five lineages, and mainly in Lineages I, II, and III which included 14/29 (48.3%), 13/38 (34.2%) and 21/45 (46.7%) MDR isolates, respectively. Ten of the 51 MDR isolates were isolated from 2016, and the remaining 41 were from 2017; By source, 13 of the 51 MDR isolates (25.5%) were isolated from diarrheal patient, four (7.8%) from the environment and 34 (66.7%) from foods (Table 1).

There were 27 MDR *C. freundii* isolates, including 9/30 (30.0%) from diarrheal patient, 17/30 (56.7%) from foods and 1/7 (14.3%) from the environment (Table 2). A total of 58 MDR *C. freundii* isolates were isolated from this study and our previous study, including 28/94 (29.8%) from diarrheal patient, 22/41 (63.7%) from foods, 7/19 (36.8%) from healthy individuals and 1/7 (14.3%) from the environment (Supplementary Table S2).

For *C. youngae,* 3 MDR isolates were isolated from this study, including 3/12 (25.0%) from diarrheal patient (Table 2) and together with 9 MDR isolates from our previous study, 5/30 (16.7%) were from diarrheal patient, 6/24 (25.0%) from foods and 1/3 (33.3%) from healthy individuals (Supplementary Table S2).

For *C. braakii,* 21 MDR isolates were isolated from this study, including 1/8 (12.5%) from diarrheal patient, 17/33 (51.5%) from foods and 3/4 (75.0%) from the environment (Table 2). Five MDR isolates were isolated from our previous study. Taken together, the MDR rate was 1/8 (12.5%) from diarrheal patient, 22/40 (55.0%) from foods and 3/4 (75.0%) from the environment (Supplementary Table S2).

### 2.3. Prevalence of ESBLs and Fluoroquinolone Resistance

Among the 128 *Citrobacter* isolates, 12 ESBL isolates were detected, which harbored *bla*_CTX-M-3_, *bla*_CTX-M-9_ or *bla*_TEM-1_, 11 of which were MDR. No isolate carried *bla_SHV_, bla_GES_, bla_PER_* or *bla_VEB_* genes.

Thirty eight (29.7%) of the 128 *Citrobacter* isolates were resistant to fluoroquinolones, including 16 *C. freundii*, 2 *C. younga*e and 20 *C. braakii* isolates, all of which were resistant to NAL (MICs ≥ 32 µg/mL); 12 resistant to CIP (MICs ≥ 2 µg/mL); and 10 resistant to LEV (MICs ≥ 8 µg/mL). By source, 27 (71.1%) of the 38 fluoroquinolones resistant isolates were from food, two from the environment and nine from diarrheal patients. These isolates were distributed among different phylogenetic lineages, 9/38 (23.7%) in Lineage I, 7/38 (1.8%) in Lineage II, 20/38 (52.6%) in Lineage III, one each in Lineage IV and V. Thirty three (86.8%) of the 38 fluoroquinolones resistant isolates were MDR (MDR ≥ 3), and 9 (23.7%) were EMBLs carrying the *bla*_CTX-M-3_, *bla*_CTX-M-9_*,* or *bla*_TEM-1_ gene (Table 3).

Twenty eight (73.7%) of the 38 NAL-resistant isolates analyzed contained mutations in the QRDR of the *gyrA* gene, with 27 having one mutation in codon 59 (Thr59Ile) and one having three mutations in codons 59, 111and 134 (Thr59Ile, Gln111Arg and Ile134Val). No mutations were found in the QRDR region of the *parC* gene (Table 3).

Of the 28 NAL-resistant isolates with *gyrA* mutations, 12 belonged to *C. freundii,* 14 to *C. braakii,* 2 to *C. younga*e. Among the 12 *C. freundii* isolates with *gyrA* mutations, six were resistant to CIP (MIC of ≥4 µg/mL), and five were resistant to LEV (MIC of ≥8 µg/mL); of the 14 *C. braakii* with *gyrA* mutations, three were resistant to CIP (MIC of 8 µg/mL), and three to LEV (MIC of ≥8 µg/mL); of the two *C. younga*e isolates with *gyrA* mutations, only one was resistant to CIP and LEV (MIC of 8 µg/mL) (Table 3).

### 2.4. Prevalence of qnrB Genes

Nineteen *Citrobacter* isolates, including 4 *C. braakii* and 15 *C. freundii* isolates, were found to harbor *qnrB* genes (including *qnrB2, qnrB9, qnrB17, qnrB76, qnrB13, qnrB93* and *qnrB94*) (Table 1). Four *C. braakii* and one *C. freundii* isolates harbored *qnrB2*, all of which were resistant to NAL, (MICs ≥ 128 µg/mL), and were MDR. *QnrB9* was found in five *C. freundii* isolates with three (HB2017053, HB2016023 and HB2017031) isolated from food and MDR, and two (HB2016004 and HB2017031) isolated from diarrheal patients and none was MDR. Among the three *qnrB9-*carrying MDR isolates, two (HB2016023 and HB2017031) were resistant to NAL (MICs ≥ 128 µg/mL). *QnrB17* was harbored in one *C. freundii* isolate (HB2017039) which was isolated from food and was not MDR. *qnrB76* was harbored by two *C. freundii* isolates with one from food and one from the environment. A variant of *qnrB76* (*qnrB76* contained a LexA binding site) was harbored by three *C. freundii* isolates*,* all of which were isolated from diarrheal patients. A variant of *qnrB13* (*qnrB13* contained a LexA binding site) was harbored by one *C. freundii* (HB2017002) isolate, which was isolated from a diarrheal patient.

Two isolates (HB2017059 and HB2017038) were found to harbor a new *qnrB* gene, both of which were isolated from food (Table 1). Sequence analysis revealed that the new *qnrB* gene harbored by HB2017059 differed from the *qnrB13* gene (GenBank accession no. EU273756.1) by one nucleotide change (a G→A change at nt85 resulting in Asp→Asn), and this new *qnrB* allele was assigned *qnrB93* (GenBank accession no.MK047606). The new *qnrB* gene harbored by HB2017038 differed from the *qnrB11* gene (GenBank accession no. EU136183.1) by seven nucleotide change, including two non-synonymous changes, and A→G change at nt430 resulted in Thr→Ala and an A→C change at nt556 resulted in Ile→Leu, and five synonymous changes, a T→G change at nt246, A→C change at nt357, G→A change at nt399, C→T change at nt468 and G→C change at nt564. This new *qnrB* allele was assigned *qnrB94* (GenBank accession no.MK047607) [26].

*qnrB* genes were predominantly harboured by Lineage I (*C. freundii*) (Table 1). When all *C. freundii* data combined, 26/53 (49.1%) Lineage I isolates carried a *qnrB* allele (Figure 2). 

### 2.5. Adherence and Cytotoxicity of Citrobacter Isolates

The 128 *Citrobacter* isolates were tested for adhesion and cytotoxicity to Hep-2 cells as done previously [10] (Table 1). Fourteen isolates (10.9%) had an adhesion index greater than 50 and were classified as high adhesion. Fifty-seven (44.5%) isolates had an adhesion index between 1 and 50 and were regarded as intermediate adhesive. Thirty seven (28.9%) isolates showed little adhesion (adhesion index of <1). The remaining 20 (15.6%) isolates showed ambivalent adhesion or no adhesion. By cytotoxicity, of the 128 isolates, 13 (10.2%) were highly cytotoxic, 40 (31.3%) intermediate cytotoxic and 75 (58.6%) non-cytotoxic.

**Figure 5:**
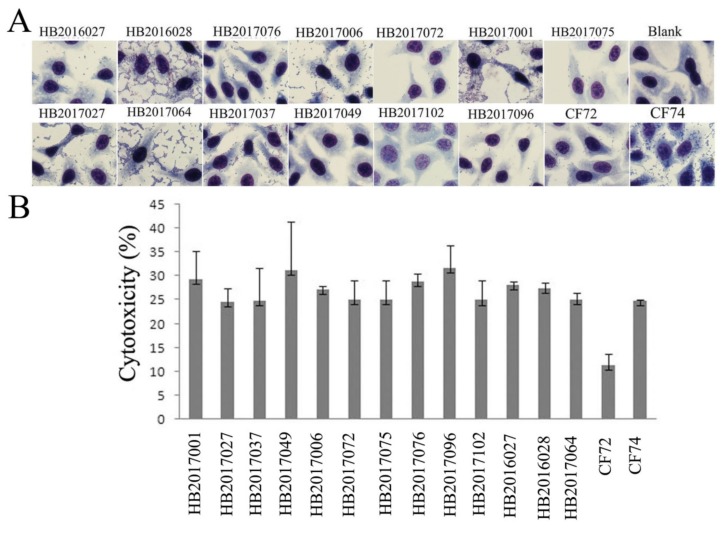
Adhesion and cytotoxicity of *Citrobacter* isolates to the human epidermoid laryngocarcinoma (Hep-2, CCC0068) cell line. (**A**) Light micrographs of the adherence patterns exhibited by the 13 highly cytotoxic *Citrobacter* isolates, and control strains CF74 and CF72. Bar: 10μm. (**B**) Cytotoxicity of the 13 highly cytotoxic *Citrobacter* isolates was measured by the amount of the LDH released after 8 h exposure by Hep-2 cells. CF72 and CF74 were control strains. CF72 was a non-cytotoxic and non- adhesive negative control, CF74 was a highly adherent and cytotoxic positive control.

Among the 14 highly adhesive isolates, four isolates released LDH more than 24%, and were considered highly cytotoxic (Figure 5); seven isolates released LDH from 19.4% to 22.9% and were considered intermediate cytotoxic; the remaining three isolates showed LDH release less than 16.5% and were likely to be non-cytotoxic (Table 1 and Supplementary Table S3).

Among the 57 intermediate adhesive isolates, seven isolates showed high cytotoxicity with more than 24% LDH released (Figure 5); 23 isolates released LDH from 18.0% to 23.5% and were intermediate cytotoxic, the remaining 27 isolates were considered to be non-cytotoxic (Table 1 and Supplementary Table S3).

Among the 37 less adhesive isolates, two showed high cytotoxicity with more than 24% LDH released (Figure 5); nine were considered intermediate cytotoxic which released LDH from 17.9% to 22.2%; the remaining 26 isolates showed LDH release less than 15.9% and are likely to be non-cytotoxic (Table 1 and Supplementary Table S3).

The 20 non adhesive isolates were also non-cytotoxic with all, except one showing intermediate cytotoxicity, releasing LDH from 0.6% to 17.1% (Table 1 and Supplementary Table S3).

**Figure 6:**
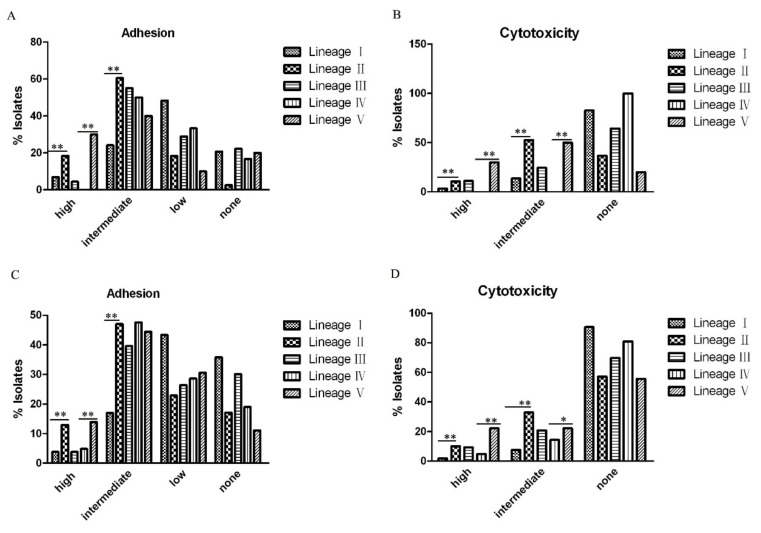
The percentage of adhesive and cytotoxic isolates in different lineages. (**A**) and (**B**) The percentage of high, intermediate, little or no adhesive or cytotoxic isolates based on the 128 *Citrobacter* isolates from this study. (**C**) and (**D**) The percentage of high, intermediate, little or no adhesive or cytotoxic isolates in different lineages based on the128 *Citrobacter* isolates from this study and 95 *Citrobacter* isolates from our previous studies [10,16]. The statistical significance between Lineages I and II or Lineages IV and V was determined by Mann-Whitney U test. *, *p* < 0.05; **, *p* < 0.01.

**Figure 7:**
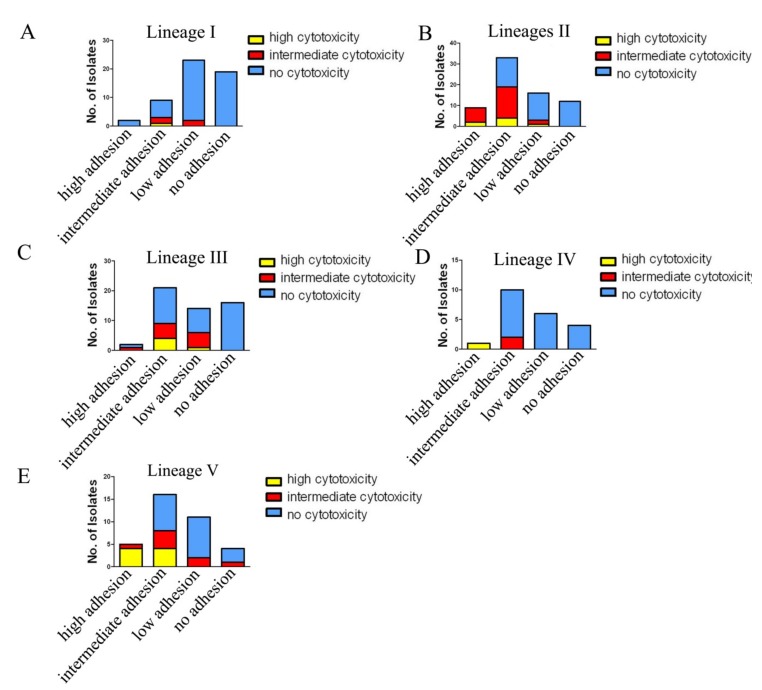
The number of adhesive and cytotoxic isolates in different lineages. The data were based on 128 isolates from this study and 95 *Citrobacter* isolates from our previous studies [10,16].

We examined any differences in adhesion and cytotoxicity between lineages. We analyzed the difference using data in this study alone (Figure 6A,C) and also using combined data with our two previous studies (Figure 6B,D). Between Lineages I and II which exclusively contained *C. freundii* isolates, Lineage II showed higher proportion of high or intermediate adhesive and cytotoxic isolates than in Lineage I and the difference is statistically significant (*p* < 0.01) (Figure 6); Between Lineage IV and V which contained only *C. youngae* isolates, the percentage of highly adhesive isolates in Lineage V was higher than in Lineage IV (*p* < 0.01) (Figure 6A,C), and the percentage of the highly or intermediate cytotoxic isolates in Lineage V was also higher than in Lineage IV (*p* < 0.05) (Figure 6B,D). When the two virulence traits were considered together, Lineages II and V had higher adhesive and cytotoxic isolates than Lineages I, III, and IV (Figure 7). 

## 3. Discussion

*Citrobacter* spp. are opportunistic pathogens that can cause diarrhea, septicemia, meningitis, and urinary tract infections, especially in immunocompromised patients [27]. Together with our previous studies [10,16], we found 25 highly cytotoxic *Citrobacter* isolates out of 271 (9.2%)*,* 15 were isolated from diarrheal patients (11.4% of diarrheal isolates), seven from foods (6.7% food isolates), two from healthy individuals (8.7%) and 1 from the environment (9.1%). The 22 highly cytotoxic *Citrobacter* isolates were distributed among the five lineages identified. However, Lineage II (*C. freundii*) and Lineage V (*C. youngae*) disproportionally contained more adhesive and more cytotoxic isolates than Lineages I, III, and IV and are likely to pose a higher risk to human health. 

### 3.1. High Genetic Diversity of Citrobacter spp. Across China and Internationally.

The 128 *Citrobacter* isolates were divided into 123 STs, displaying high genetic diversity. We compared our STs with 268 STs from the *Citrobacer* MLST database and found 22 STs in this study were present in the database with isolates from other countries or regions, or from different sources. Among these 22 STs, ST1 contained isolates from diarrheal patients, healthy individuals, animals, insects and environment in our previous study [9]; ST12 contained isolates from a rectal swab (Israel in 2009) and a diarrheal patient (China in 2015) [16]; ST17 contained isolates from a skin necrosis, urine (Poland, 2012), two rectal swabs (Latvia, 2008 and 2009), and two diarrheal patients (China in 2014) [16]; ST163 and ST169 contained isolates from water (Canada in 2015); ST161 contained isolates from water (Canada in 2015) and a diarrheal patient (China in 2015) [16]; ST85, ST183, ST214, ST216, ST217, ST225, and ST237 each contained isolates from diarrheal patient in our previous studies [10,18]; ST187 and ST219 each contained isolates from healthy individuals in our previous study [16]; ST258 and ST260 each contained isolates from the Netherlands. Our analysis by clonal complexes which allowed one to examine closely related STs, found 17 *C. freundii* CCs containing at least 2 STs per CC contained isolates from different countries with one CC present in 5 different countries. For *C. youngae* and *C. braakii,* fewer CCs were identified, and narrower geographic distribution was found. However, there was no information from other countries for comparison for these latter two species. Thus, some *Citrobacter* STs and CCs are likely to be widely present in fecal, food, and other reservoirs and spread in different countries or regions. However, there are very limited reports of *Citrobacter* spp. from different sources or geographical regions. A recent study in companion animals reported nosocomial dissemination of *C. freundii* strains resistant to extended-spectrum cephalosporins [28]. Our studies underscore the need for further study to understand the genetic diversity, virulence and antibiotic resistance and their risks to human health.

### 3.2. Association of C. freundii Lineage II and C. youngae Lineage V with Higher Adhesion and Cytotoxicity

The clustering of highly adhesive and cytotoxic isolates in specific lineages is most interesting as it suggests that different lineages have different levels of virulence and/or may cause different types of diseases. Combining this study with our previous studies [10,16], there were nine highly cytotoxic *C. freundii* isolates, of which six were isolated from diarrheal patients, two from foods, and one from the environment. Among these six highly cytotoxic *C. freundii* isolates from diarrheal patients, five isolates were clustered in Lineage II and one in Lineage I, suggesting that Lineage II is more likely to cause diarrheal disease. *C. freundii*, as the most common *Citrobacter* species, has caused gastroenteritis associated outbreaks [3]. However, despite reports of different virulence factors involved [9,10], little is known of its pathogenic mechanisms and there have been no means that can differentiate strains that can cause diarrheal disease from those of harmless colonizers. The identification of Lineage II as more likely to cause diarrhea provided an avenue to further identify virulence genes involved and to determine whether Lineage II is more likely to cause other infections.

Similarly, combining with our previous studies [10,16], seven of the eight highly cytotoxic *C. youngae* strains fell into Lineage V, of which four were isolated from diarrheal patients, suggest that Lineage Ⅴ is likely to cause diarrheal disease. However, *C. younage* had not been recognized as a diarrheal pathogen and Lineage V should be further investigated for their potential to cause diarrheal disease.

*C. braakii* has been isolated from foods, hospital infections and UTIs [14], and acute peritonitis patients [7,8]. In our previous study, we isolated *C. braakii* isolates from food source, but not human fecal samples [10]. In this study, we obtained 45 *C. braakii* isolates from food, environment and diarrheal patients with two highly adhesive and five highly cytotoxic isolates. Three of the five highly cytotoxic *C. braakii* isolates were isolated from diarrheal patients, and it remains to be determined whether *C. braakii* can cause diarrhea.

### 3.3. Higher Prevalence of Multidrug Resistance in C. braakii Isolates and Citrobacter Isolates from Food Sources

MDR has been reported in *Citrobacter* isolates, especially *C. freundii* [17]. Considering isolates from this study only, 58 of the 128 isolates were MDR, mainly distributed among Lineages I to III. When combined with our previous studies [10,16], 96 of the 271 *Citrobacter* isolates (35.4%) were MDR, including 58 *C. freundii* isolates (36.0% of *C. freundii* isolates, Lineages I and II)*,* 12 *C. youngae* isolates (21.1% of *C. youngae* isolates, Lineages IV and V) and 26 *C. braakii* isolates (49.0% of *C. braakii* isolates, Lineage III). The difference in the prevalence of MDR between *C. youngae* and *C. braakii* is statistically significant (p < 0.01).

Among the 96 MDR isolates, 34 were isolated from diarrheal patients (25.8% of diarrheal isolates), 50 from foods (47.6% of the food isolates), four from the environment (36.4%) and eight from healthy individuals (34.8%). Interestingly MDR were more prevalent among food isolates. Since most of the food source was related to meat or meat products [10,16], the MDR may have been a result of extensive use of antibiotics in animals. Moreover, four highly cytotoxic strains (11.8%) were found in 34 MDR isolates from diarrheal patients, highlighting the combined increased risk of high cytotoxicity and MDR of *Citrobacter* to human health.

### 3.4. Carriage of ESBL Genes by Citrobacter spp. was Relatively Low 

ESBL producing *Enterobacteriaceae* has been a major challenge in infection control [29,30]. Previous studies have reported that *Citrobacter* isolated from human clinical infections that carried ESBL genes varied from 5.6% to 67.5%, including *bla*_TEM__−1,_ *bla*_SHV-12,_ *bla*_CTX__−M__−15_and *bla*_CTX__−M_ [3,28]. In our studies, including our previous studies [10,16], we found 2.6% of *Citrobacter* isolates carried the *bla*_CTX__−M-9_ gene, 1.1% carried the *bla*_CTX__−M-3_ gene, 3.7% carried the *bla*_TEM-1_ gene, and none carried the *bla*_SHV_ gene. The ESBL carrying *Citrobacter* isolates consisted of 12 *C. freundii* isolates (7.5% of *C. freundii* isolates) and four *C. braakii* isolates (7.5% of *C. braakii* isolates). The four ESBL carrying *C. braakii* isolates were isolated from foods. Three of the 12 ESBLs carrying *C. freundii* isolates were isolated from diarrheal patients (2.3% of diarrheal isolates), eight from foods (7.6%) and one from healthy individuals (4.3%). In comparison to other reports, ESBL carriage is lower in our isolates. The isolates of our previous two studies [10,16] were from the south of China (Anhui province), while isolates of this study were from northern China (Hebei Province), suggesting likely low prevalence of ESBLs in *Citrobacter* spp. In China. However, other regions of China would need to be sampled to obtain a more comprehensive picture.

### 3.5. Higher Prevalence of Quinolone Resistance in Lineages I and III of Citrobacter spp with Multiple Mechanism of Resistance Detected

The prevalence of quinolone resistance varied among the lineages. Lineage III (*C. braakii*) had the highest proportion of resistance isolates (52.6%), followed by Lineage I (*C. freundii*) with 23.7%. We analyzed the carriage of potential quinolone resistance genes or mutations which include both the QRDR of *gyrA* and *parC* associated resistance, and PMQR genes mediated resistance [22]. *Citrobacter* isolates with mutations in the QRDR of *gyrA, including* Thr83Ile and Asp87Asn have shown reduced susceptibility to fluoroquinolones [22,23]. In our previous study [18], four (66.7%) of the six ciprofloxacin-resistant *C. freundii* isolates were found to have mutations in codons 59, 111, and/or 134 in the QRDR region of the *gyrA* gene, namely, Thr59Ile, Gln111Arg, and Ile134Val. In this study, 28 ciprofloxacin-resistant isolates carried mutations in the QRDR of *gyrA* with 27 having one mutation, Thr59Ile and one having three mutations, Thr59Ile, Gln111Arg and Ile134Val. However, it should be noted that these three mutations have not been experimentally confirmed whether they affect fluoroquinolone resistance.

For PMQR gene-mediated resistance, 14.0% of our *Citrobacter* isolates carried a *qnr* gene, and 5.5% of *Citrobacter* isolates carried the *aac(6’)-Ib-cr* gene. Our results contrast previous reports of high prevalence of *qnr* and *aac(6′)-Ib-cr* genes from clinical infections in China at 72.8% and 68.9% in *C. freundii* isolates; and 42.9% and 42.9% in *C. braakii* isolates, respectively from the study of Zhang et al. [29], and 63.3% and 26.7% in *C. freundii* from the study of Yang *et al.* [31]. This difference could be due to different regions of China or the sources of samples. 

### 3.6. Citrobacter spp. Carried Many Variants of the qnrB Gene with C. freundii Lineage I as the Main Reservoir

*qnrB* is known to be harbored by *Citrobacter* [26]. Surprisingly our *Citrobacter* isolates harboured many *qnrB* variants with 11 *qnrB* allelic variants found, including *qnrB2, qnrB9, qnrB13, qnrB16, qnrB17, qnrB63, qnrB76, qnrB77, qnrB92, qnrB93* and *qnrB94.* Interestingly, although only 17% (28 of 161) of the *C. freundii* isolates harbored *qnrB* genes, all except one *qnrB* positive isolate belonged to Lineage I, suggesting that Lineage I is a main reservoir of *qnrB* genes. However, carriage of *qnrB* gene does not always confer high level of quinolone resistance [29]. Only two *qnrB9*-carrying *C. freundii* isolates in Lineage I were resistant to quinolones, while other *qnrB*-carrying *C. freundii* isolates in Lineage I were susceptible to NAL, CIP and LEV. The only *qnrB* carrying Lineage II isolate is quinolones resistant. Four *qnrB*-carrying *C. braakii* isolates were resistant to NAL (MICs ≥ 128 µg/mL). These results suggest that *Citrobacter* isolates carrying different *qnrB* alleles may have different levels of quinolone resistance.

*QnrB* has a growing number of allelic variants [32]. In our previous study, we found a variant of *qnrB77* (a *qnrB77* contained a LexA binding site) and a new *qnrB* allele (*qnrB92*) [10,16]. In this study, we found a variant of *qnrB76* (a *qnrB76* contained a LexA binding site), a variant of *qnrB13* (a *qnrB13* contained a LexA binding site), and two new *qnrB* alleles, *qnrB93* and *qnrB94*.

## 4. Conclusions

We analyzed 128 *Citrobacter* isolates (67 *C. freundii*, 16 *C. younga*e and 45 *C. braakii* isolates) from human diarrheal patients, foods and environment in Shijiazhuang, Hebei Province in the north of China. This study expands the genetic diversity observed from our previous studies of human and food isolates from South China [10,16]. The isolates showed high diversity with 123 STs of which 101 were novel STs. Only 22 STs (17.9%) were found from different sources and/or geographic regions. Phylogenetic analysis divided the 128 isolates into five lineages. Lineages I and II contained *C. freundii* isolates, while Lineages IV and V contained *C. youngae* isolates and Lineage III contained all *C. braakii* isolates. 

The 51 MDR isolates were mainly distributed in Lineage I (*C. freundii*) and Lineage III (*C. braakii*) with 48.3% and 46.7% of the isolates in these lineages being MDR, respectively. Food isolates were also more likely to be MDR. Combining data with our previous studies [10,16], we found that the prevalence of quinolone resistance was highest in Lineage I (*C. freundii*) and Lineage III (*C. braakii*). *Citrobacter spp*. carried many variants of the *qnrB* gene with the carriage by *C. freundii* Lineage I isolates being the highest. Surprisingly, the prevalence of ESBL genes in *Citrobacter spp*. is relatively low.

There were 22 highly cytotoxic *Citrobacter* isolates, which were preferentially distributed in *C. freundii* Lineage II and *C. youngae* Lineage V, suggesting that these two lineages are more virulent than others, and thus, more likely to cause disease. This study has shed more light on the genetic diversity, pathogenicity and antibiotic resistance of *Citrobacter.*

## 5. Methods

### 5.1. Citrobacter Isolates

128 *Citrobacter* spp. isolates were isolated from 50 diarrheal patients, 11 environment and 67 food samples in Shijiazhuang Hebei Province, China from 2016 to 2017. 50 diarrheal patient fecal samples included 30 *C. freundii,* eight *C. braakii* and 12 *C. youngae* isolates, and four of the 50 diarrheal patient fecal samples harbored *norovirus* (HB2016002 and HB2017022) or *Vibrio parahaernolyticus* (HB2016004 and HB2016036); the 11 environment isolates had seven *C. freundii* and four *C. braakii* which were isolated from food processing place; 67 food isolates included 30 *C. freundii,* 33 *C. braakii* and four *C. youngae* isolates which were isolated from chicken, pork, duck and vegetables (Supplementary Table S4). The species identity of each isolate was confirmed using API 20E test strips (bioMérieux, La Balme les Grottes, France). Bacterial culture media and conditions were as previously described [10].

### 5.2. MLST and Phylogenetic Analysis

The *Citrobacter* MLST scheme (http://pubmlst.org/*cfreundii*/) was used. PCR amplification and sequencing were carried out using published primers as described previously [10]. SeqMan 7.0 software was used to analyze the sequences.

### 5.3. Antimicrobial Susceptibility Testing

Antimicrobial susceptibility testing was carried out as previously described [10,33]. Minimum inhibitory concentration (MIC) was interpreted in 22 antibiotics, including ampicillin, cefotaxime, ceftazidime, cefepime, cefoxitin, ceftiofur Sodium, aztreonam, imipenem, meropenem, nalidixicacid, ciprofloxacin, levofloxacin, gentamicin, amikacin, streptomycin, kanamycin, tetracycline, doxycycline, chloramphenicol, trimethoprim/sulfamethoxazole, sulfafurazole and azithromycin (Table 2). *E. coli* ATCC 25922 and *Pseudomonas aeruginosa* ATCC 27853 were used as quality controls for MICs.

### 5.4. PCR Amplification and Sequencing.

Detection of *bla*_TEM_, *bla*_SHV_, *bla*_CTX-M-1_, *bla*_CTX-M-2_, *bla*_CTX-M-8_, *bla*_CTX-M-9_**_,_** *bla*_GES_, *bla*_PER_ and *bla*_VEB_, *qnrA, qnrB, qnrS, qnrC, qnrD, aac(6')-Ib-cr* and *qepA* genes were carried out as previously described [10,34,35,36,37,38,39]. 

### 5.5. In Vitro Adhesion and Cytotoxicity Assays.

The human epidermoid laryngocarcinoma (HEp-2, CCC0068) cell line was obtained from the cell resource center at Peking Union Medical College. HEp-2 cell line has been used as a human cell line to study bacteria-host interactions in many studies [40,41,42]. Note that Hep-2 cell line may have been a misidentified cell line, raising concerns of relevance to laryngeal cancer research [43], but the issue is not relevant here. *In vitro* adhesion to host cells was performed as previously described [10]. The mean number of bacteria per HEp-2 after examination of 10 visual fields was determined and was used as an adhesion index (<1; >1 and <50; >50) [10]. 

The lactate dehydrogenase (LDH) released by the HEp-2 cells was determined as previously described [10]. All experiments were performed three times in duplicate.

### 5.6. Statistical Analysis.

SPSS software version 13.0 (SPSS Inc., Chicago, IL, USA) was used to conduct all statistical comparisons. A nonparametric test (Mann–Whitney U-test) and chi-square test were used to compare the different groups for statistical significance. *p*-value of ≤ 0.05 (2-tailed) was considered to be statistically significant. 

### 5.7. Ethics Approval and Consent to Participate

This study was reviewed and approved by the ethics committee of the National Institute for Communicable Disease Control and Prevention, the Chinese CDC. Human fecal specimens were acquired with the written informed consent of the diarrheal patients with the approval of the ethics committee of the National Institute for Communicable Disease Control and Prevention, according to the medical research regulations of Ministry of Health (ICDC-2016004).

## Figures and Tables

**Table 1:** Adherence, cytotoxicity, multidrug-resistant and Genotypes of 128 *Citrobacter* Isolates.

Clusters and Species	Isolates	Year	Source	STs	Adhesion	LDH	NDR	ESBLs	*qnr*
Lineage Ⅰ	HB2016004	2016	D	17	***	12.4 ± 0.4	2		*qnrB9*
*C. freundii*	HB2016019	2016	F	284	+/-	6.3 ± 2.2	9		*qnrB9*
	HB2016023	2016	F	288	*	8.9 ± 0.2	9	*bla*_CTX-M-3_, *bla*_TEM-1_	*aac(6')-Ib-cr,qnrB9*
	HB2016024	2016	F	289	*	20.5 ± 1.3	3		
	HB2016034	2016	F	260	*	5.0 ± 1.4	7		*qnrS1*
	HB2016036	2016	D	298	+/-	0.6 ± 0.2	2		
	HB2017002	2017	D	300	+/-	10.3 ± 1.8	1	*bla*_CTX-M-9_	*qnrS1,qnrB13 include the LexA binding site*
	HB2017003	2017	D	301	*	8.9 ± 0.8	2		
	HB2017004	2017	D	85	*	12.3 ± 2.2	2		
	HB2017006	2017	D	303	**	27.3 ± 0.6	2		*qnrB76* include the LexA binding site
	HB2017009	2017	D	304	*	11.9 ± 0.6	4		*qnrB76* include the LexA binding site
	HB2017012	2017	D	306	*	7.1 ± 0.4	1		*qnrB76* include the LexA binding site
	HB2017016	2017	D	309	*	15.0 ± 1.7	1		
	HB2017017	2017	D	1	*	15.9 ± 1.0	2		
	HB2017026	2017	D	313	**	17.4 ± 1.1	6		*aac(6')-Ib-cr*
	HB2017031	2017	D	318	+/-	12.7 ± 0.2	2		*qnrB9*
	HB2017033	2017	E	320	**	19.1 ± 3.2	0		*qnrB76*
	HB2017036	2017	E	322	**	14.1 ± 0.8	3		
	HB2017038	2017	F	324	*	12.4 ± 8.9	4		*qnrB94*
	HB2017039	2017	F	325	*	19.8 ± 5.7	2		*qnrB17*
	HB2017040	2017	F	326	*	6.7 ± 1.4	8		
	HB2017042	2017	F	328	-	8.9 ± 7.8	9	*bla*_TEM-1_	
	HB2017045	2017	F	331	*	14.3 ± 1.8	7	*bla*_CTX-M-9_, *bla*_TEM-1_	*qnrS1*
	HB2017052	2017	F	337	**	15.5 ± 1.5	2		
	HB2017053	2017	F	338	**	21.6 ± 2.5	4		*qnrB9*
	HB2017054	2017	F	339	***	16.5 ± 3.6	2		*qnrB76*
	HB2017059	2017	F	343	*	12.8 ± 0.4	7	*bla*_CTX-M-3_ *, bla*_CTX-M-9_	*qnrB93*
	HB2017060	2017	F	344	+/-	11.9 ± 1.6	7	*bla*_CTX-M-9_, *bla*_TEM-1_	
	HB2017061	2017	F	1	**	17.3 ± 2.4	2		
Lineage Ⅱ	HB2016001	2016	D	269	**	11.0 ± 2.1	1		
*C. freundii*	HB2016002	2016	D	216	*	10.4 ± 0.2	3		
	HB2016003	2016	D	270	**	15.6 ± 1.1	1		
	HB2016006	2016	E	272	**	9.3 ± 2.8	1		
	HB2016008	2016	F	274	***	22.7 ± 7.3	8	*bla*_TEM-1_	*aac(6')-Ib-cr,qnrB2*
	HB2016010	2016	E	276	*	8.4 ± 1.9	1		
	HB2016011	2016	F	100	***	22.6 ± 3.0	1		
	HB2016012	2016	F	277	**	16.9 ± 1.5	2		
	HB2016013	2016	F	278	**	15.3 ± 3.9	3		
	HB2016017	2016	F	282	***	21.4 ± 7.3	1		
	HB2016018	2016	F	283	+/-	9.2 ± 1.5	2		
	HB2017001	2017	D	169	***	29.4 ± 5.8	2		
	HB2017008	2017	D	12	*	13.6 ± 0.7	5		
	HB2017011	2017	D	163	**	15.8 ± 0.7	7	*bla*_TEM-1_	*aac(6')-Ib-cr*
	HB2017013	2017	D	307	**	6.8 ± 0.3	1		
	HB2017014	2017	D	308	**	18.0 ± 13.5	1		
	HB2017018	2017	D	125	**	21.5 ± 7.3	2		
	HB2017019	2017	D	217	**	14.6 ± 1.4	1		
	HB2017020	2017	D	310	***	22.9 ± 0.9	1		
	HB2017022	2017	D	311	**	20.2 ± 1.3	2		
	HB2017023	2017	D	219	**	18.0 ± 3.4	3		
	HB2017024	2017	D	150	**	21.2 ± 1.0	1		
	HB2017025	2017	D	312	*	8.0 ± 5.0	3		
	HB2017027	2017	D	314	***	24.7 ± 2.7	4		
	HB2017030	2017	D	317	**	20.2 ± 3.0	1		
	HB2017032	2017	D	319	**	19.7 ± 1.3	5		
	HB2017034	2017	E	100	***	19.4 ± 2.5	1		
	HB2017035	2017	E	321	**	21.8 ± 5.7	1		
	HB2017037	2017	E	323	**	24.8 ± 6.8	1		
	HB2017041	2017	F	327	*	5.4 ± 1.6	1		
	HB2017043	2017	F	329	*	20.0 ± 0.6	1		
	HB2017046	2017	F	332	**	22.9 ± 7.0	2		
	HB2017047	2017	F	333	**	23.5 ± 5.0	6		
	HB2017049	2017	F	335	*	31.2 ± 10.2	2		
	HB2017051	2017	F	214	**	21.0 ± 4.4	2		
	HB2017055	2017	F	161	**	18.2 ± 3.1	6		
	HB2017056	2017	F	340	**	19.2 ± 3.4	4		
	HB2017057	2017	F	341	**	20.3 ± 3.3	4		
Lineage Ⅲ	HB2016015	2016	F	280	+/-	3.4 ± 0.7	5		*qnrS1*
*C. braakii*	HB2016032	2016	E	295	**	4.2 ± 4.1	5		
	HB2016033	2016	F	296	**	5.3 ± 3.2	2		
	HB2016035	2016	F	297	*	11.1 ± 2.2	6		*aac(6')-Ib-cr,qnrB2*
	HB2017044	2017	F	330	**	20.4 ± 5.5	2		
	HB2017048	2017	F	334	*	20.6 ± 4.0	2		
	HB2017062	2017	F	345	*	14.8 ± 1.9	2		
	HB2017068	2017	F	351	**	12.5 ± 8.9	2		
	HB2017070	2017	D	353	*	9.5 ± 0.4	2		
	HB2017071	2017	D	354	*	22.2 ± 6.9	2		
	HB2017072	2017	D	355	**	25.2 ± 4.0	1		
	HB2017074	2017	D	356	***	14.9 ± 7.4	2		
	HB2017075	2017	D	357	**	25.2 ± 4.0	1		
	HB2017076	2017	D	358	**	28.9 ± 1.6	4		
	HB2017077	2017	D	357	**	13.6 ± 0.2	1		
	HB2017078	2017	D	359	+/-	13.7 ± 5.7	1		
	HB2017079	2017	E	360	*	15.5 ± 2.0	3		
	HB2017081	2017	E	362	**	17.7 ± 0.8	2		
	HB2017082	2017	F	363	**	13.1 ± 1.9	1		
	HB2017083	2017	F	364	**	10.8 ± 0.6	1		
	HB2017084	2017	F	365	**	13.9 ± 2.0	3		
	HB2017087	2017	F	367	*	3.5 ± 4.0	8		*aac(6')-Ib-cr, qnrB2*
	HB2017089	2017	F	369	+/-	11.7 ± 9.7	2		
	HB2017090	2017	F	370	+/-	4.5 ± 1.7	8		
	HB2017091	2017	F	371	-	4.7 ± 0.1	3		
	HB2017092	2017	F	372	*	11.7 ± 10.7	4		
	HB2017093	2017	F	373	+/-	6.5 ± 2.0	2		
	HB2017094	2017	F	81	+/-	9.8 ± 5.6	3		
	HB2017095	2017	F	225	**	6.2 ± 2.5	6	*bla*_CTX-M-9_	*qnrB2*
	HB2017096	2017	F	374	**	31.7 ± 4.8	6	*bla*_CTX-M-3_	
	HB2017097	2017	F	375	**	17.5 ± 1.9	0		
	HB2017098	2017	F	376	+/-	14.2 ± 6.5	1		
	HB2017099	2017	E	297	+/-	17.1 ± 1.1	6		*aac(6')-Ib-cr*
	HB2017100	2017	F	377	*	17.9 ± 2.2	2		
	HB2017101	2017	F	378	***	21.1 ± 5.1	2		
	HB2017102	2017	F	379	*	25.0 ± 4.2	3		
	HB2017103	2017	F	380	*	0.7 ± 0.4	4		
	HB2017104	2017	F	381	+/-	14.0 ± 1.7	3		
	HB2017105	2017	F	382	**	13.9 ± 2.9	9	*bla*_TEM-1_	*aac(6')-Ib-cr*
	HB2017106	2017	F	383	**	18.6 ± 0.3	4		
	HB2017107	2017	F	384	*	18.5 ± 1.1	7		
	HB2017108	2017	F	385	**	13.2 ± 3.1	6	*bla*_CTX-M-9_	*aac(6')-Ib-cr, qnrB2*
	HB2017109	2017	F	386	*	20.4 ± 2.4	1		
	HB2017110	2017	F	375	**	18.0 ± 3.0	0		
	HB2017111	2017	F	387	**	18.0 ± 0.8	2		
Lineage Ⅳ	HB2016029	2016	D	292	+/-	6.5 ± 4.8	1		
*C. youngae*	HB2017007	2017	D	237	*	8.4 ± 2.3	8		*aac(6')-Ib-cr*
	HB2017021	2017	D	74	*	10.0 ± 0.5	3		
	HB2017029	2017	D	316	**	16.5 ± 2.5	2		
	HB2017067	2017	D	350	**	15.3 ± 2.7	1		
	HB2017086	2017	F	258	**	7.0 ± 5.1	2		
Lineage Ⅴ	HB2016026	2016	D	183	**	17.2 ± 0.9	1		
*C. youngae*	HB2016027	2016	D	291	**	28.3 ± 0.5	1		
	HB2016028	2016	D	187	***	27.4 ± 1.2	0		
	HB2016031	2016	F	294	**	18.7 ± 2.7	2		
	HB2017063	2017	D	346	*	18.7 ± 1.5	2		
	HB2017064	2017	D	347	***	25.2 ± 1.4	0		
	HB2017065	2017	D	348	***	20.4 ± 4.9	7		
	HB2017066	2017	D	349	-	19.5 ± 3.2	0		
	HB2017069	2017	F	352	+/-	16.2 ± 14.6	2		
	HB2017085	2017	F	366	**	22.1 ± 6.5	1		

Adhesion index: ***, >50; **, >1 and <50; *, <1; +/-, ambivalent or no adhesion; -, no adhesion. LDH (% ± SD): The lactate dehydrogenase released from Hep-2 cells; STs, sequence types; NDR, number of drugs resistance; D, F and E, isolates from diarrheal patients, foods and environment. ESBLs: extended-spectrum β-lactamase.

**Table 2:** Prevalence of resistance to different antibiotics by species and source.

Antibiotic	*C. freundii* (n = 67) Resistance (%)			*C. youngae* (n = 16) Resistance (%)			*C.braakii* (n = 45) Resistance (%)		
	D (n = 30)	F (n = 30)	E (n = 7)	D (n = 12)	F (n = 4)	E (n = 0)	D (n = 8)	F (n = 33)	E (n = 4)
PENICILLINS									
Ampicillin	15 (50.0)	23 (76.7)	1 (14.3)	5 (41.7)	3 (75.0)	0 (0)	3 (37.5)	16 (48.5)	4 (100.0)
CEPHALOSPORINS									
Cefotaxime	6 (20.0)	12 (40.0)	0 (0)	1 (8.3)	0 (0)	0 (0)	0 (0)	7 (21.2)	2 (50.0)
Ceftazidime	3 (10.0)	3 (10.0)	0 (0)	1 (8.3)	0 (0)	0 (0)	0 (0)	1 (3.0)	0 (0)
Cefepime	0 (0)	6 (20.0)	0 (0)	0 (0)	0 (0)	0 (0)	0 (0)	1 (3.0)	0 (0)
Cefoxitin	28 (93.3)	29 (96.7)	6 (85.7)	8 (66.7)	3 (75.0)	0 (0)	7 (87.5)	29 (87.9)	4 (100.0)
Ceftiofur Sodium	3 (10.0)	11 (36.7)	0 (0)	0 (0)	0 (0)	0 (0)	0 (0)	7 (21.2)	1 (25.0)
MONOBACTAMS									
Aztreonam	2 (6.7)	3 (10.0)	0 (0)	1 (8.3)	0 (0)	0 (0)	0 (0)	5 (15.2)	0 (0)
CARBAPENEMS									
Imipenem	0 (0)	1 (3.3)	0 (0)	0 (0)	0 (0)	0 (0)	0 (0)	1 (3.0)	0 (0)
Meropenem	0 (0)	3 (10.0)	0 (0)	0 (0)	0 (0)	0 (0)	0 (0)	0 (0)	0 (0)
QUINOLONES									
Nalidixicacid	4 (13.3)	12 (40.0)	0 (0)	2 (16.7)	0 (0)	0 (0)	3 (37.5)	15 (45.5)	2 (50.0)
Ciprofloxacin	2 (6.7)	4 (13.3)	0 (0)	1 (8.3)	0 (0)	0 (0)	0 (0)	3 (9.1)	0 (0)
Levofloxacin	1 (3.3)	4 (13.3)	0 (0)	1 (8.3)	0 (0)	0 (0)	0 (0)	3 (9.1)	0 (0)
AMINOGLYCOSIDES									
Gentamicin	0 (0)	7 (23.3)	0 (0)	0 (0)	0 (0)	0 (0)	0 (0)	2 (6.1)	0 (0)
Amikacin	0 (0)	0 (0)	0 (0)	0 (0)	0 (0)	0 (0)	0 (0)	0 (0)	0 (0)
Streptomycin	2 (6.7)	11 (36.7)	1 (14.3)	2 (16.7)	0 (0)	0 (0)	0 (0)	8 (24.2)	2 (50.0)
Kanamycin	0 (0)	3 (10.0)	0 (0)	1 (8.3)	0 (0)	0 (0)	0 (0)	1 (3.0)	0 (0)
TETRACYCLINES									
Tetracycline	7 (23.3)	15 (50.0)	0 (0)	3 (25.0)	0 (0)	0 (0)	1 (12.5)	17 (51.5)	1 (25.0)
Doxycycline	2 (6.7)	9 (30.0)	0 (0)	2 (16.7)	0 (0)	0 (0)	1 (12.5)	12 (36.4)	1 (25.0)
PHENICOLS									
Chloramphenicol	6 (20.0)	11 (36.7)	0 (0)	2 (16.7)	0 (0)	0 (0)	0 (0)	7 (21.2)	2 (50.0)
SULFONAMIDES									
Trimethoprim/Sulfamethoxazole	4 (13.3)	13 (43.3)	0 (0)	2 (16.7)	1 (25.0)	0 (0)	0 (0)	11 (33.3)	2 (50.0)
Sulfafurazole	3 (10.0)	12 (40.0)	0 (0)	3 (25.0)	0 (0)	0 (0)	0 (0)	9 (37.3)	2 (50.0)
MACROLIDES									
Azithromycin	1 (3.3)	6 (20.0)	0 (0)	0 (0)	0 (0)	0 (0)	0 (0)	3 (9.1)	0 (0)
MDR	9 (30.0)	17 (56.7)	1 (14.3)	3 (25.0)	0 (0)	0 (0)	1 (12.5)	17 (51.5)	3 (75.0)

D, diarrheal patients; F, foods; E, environment. MDR: With resistance to at least one antibiotic of three or more distinct classes (MDR ≥ 3).

**Table 3:** Quinolone resistant *Citrobacter* isolates and the presence of quinolone resistance genes and alterations in the *gyrA* gene.

Isolates	Species	Year	Source	ST	NDR	NAL	CIP	LEV	PMQR	*gyrA* Position
HB2016008	*C. freundii*	2016	F	274	8	>128	4		*aac(6’)-Ib-cr,qnrB2*	Thr59Ile
HB2016017	*C. freundii*	2016	F	282	1	>128				Thr59Ile
HB2017011	*C. freundii*	2017	D	163	7	>64	16	8	*aac(6’)-Ib-cr*	Thr59Ile
HB2017018	*C. freundii*	2017	D	125	2	>128				Thr59Ile
HB2017027	*C. freundii*	2017	D	314	4	>64				No mutation
HB2017055	*C. freundii*	2017	F	161	6	>128				Thr59Ile
HB2017056	*C. freundii*	2017	F	340	4	>128				Thr59Ile
HB2016019	*C. freundii*	2016	F	284	9	>128		8	*qnrB9*	Thr59Ile
HB2016023	*C. freundii*	2016	F	288	9	>128	32	16	*aac(6’)-Ib-cr, qnrB9*	Thr59Ile
HB2016024	*C. freundii*	2016	F	289	3	>128	4	8		Thr59Ile
HB2016034	*C. freundii*	2016	F	260	7	32			*qnrS1*	No mutation
HB2017026	*C. freundii*	2017	D	313	6	>128	4		*aac(6’)-Ib-cr*	Thr59Ile
HB2017040	*C. freundii*	2017	F	326	8	>128				Thr59Ile
HB2017042	*C. freundii*	2017	F	328	9	>128	8	16		Thr59Ile
HB2017045	*C. freundii*	2017	F	331	7	64			*qnrS1*	No mutation
HB2017060	*C. freundii*	2017	F	344	7	>128				No mutation
HB2016015	*C. braakii*	2016	F	280	5	>128			*qnrS1*	Thr59Ile
HB2016033	*C. braakii*	2016	F	296	2	>128				Thr59Ile
HB2016035	*C. braakii*	2016	F	297	6	>128	8	8	*aac(6’)-Ib-cr, qnrB2*	Thr59Ile
HB2017070	*C. braakii*	2017	D	353	2	>64				Thr59Ile
HB2017076	*C. braakii*	2017	D	358	4	>128				Thr59Ile
HB2017078	*C. braakii*	2017	D	359	1	>64	2			No mutation
HB2017079	*C. braakii*	2017	E	360	3	>128				Thr59Ile
HB2017084	*C. braakii*	2017	F	365	3	>64				Thr59Ile
HB2017087	*C. braakii*	2017	F	367	8	>128			*aac(6’)-Ib-cr, qnrB2*	Thr59Ile
HB2017090	*C. braakii*	2017	F	370	8	>128	8	>16		Thr59Ile
HB2017091	*C. braakii*	2017	F	371	3	>128				Thr59Ile
HB2017092	*C. braakii*	2017	F	372	4	64				No mutation
HB2017095	*C. braakii*	2017	F	225	6	>128			*qnrB2*	No mutation
HB2017099	*C. braakii*	2017	E	297	6	>128	8	8	*aac(6’)-Ib-cr*	Thr59Ile
HB2017102	*C. braakii*	2017	F	379	3	32				No mutation
HB2017103	*C. braakii*	2017	F	380	4	>128				Thr59Ile
HB2017105	*C. braakii*	2017	F	382	9	>128	8	16	*aac(6’)-Ib-cr*	No mutation
HB2017106	*C. braakii*	2017	F	383	4	32				No mutation
HB2017107	*C. braakii*	2017	F	384	7	>128				Thr59Ile
HB2017108	*C. braakii*	2017	F	385	6	>128			*aac(6’)-Ib-cr, qnrB2*	Thr59Ile
HB2017007	*C. youngae*	2017	D	237	8	>64	8	8	*aac(6’)-Ib-cr*	Thr59Ile
HB2017065	*C. youngae*	2017	D	348	7	>128				Thr59Ile, Gln111Arg, Ile134Val

NAL, nalidixicacid; CIP, ciprofloxacin; LEV, levofloxacin. NDR, number of drugs resistant to. PMQR, plasmid-mediated quinolone resistance (PMQR) genes.

## Supplementary Materials

The following are available online at https://www.mdpi.com/2076-0817/9/3/195/s1, Figure S1, Phylogenetic relationships as determined by MLST data for the 123 *C. freundii* isolates from this study and two previous studies; Table S1, List of clonal complexes of *Citrobacter* isolates from this study and the public MLST database^#^; Table S2, Prevalence of MDR isolates in different *Citrobacter* species; Table S3, Adherence and Cytotoxicity in 128 *Citrobacter* Isolates; Table S4, Source, drugs resistance, Genotypes and Antibiotic resistance phenotype of 128 *Citrobacter* Isolates.
