# Supplementary material for: Lineage, Antimicrobial Resistance and Virulence of Citrobacter spp
Source: Pathogens. 2020 Mar 6;9(3):195. doi: 10.3390/pathogens9030195 (PMC7157202; doi:10.3390/pathogens9030195)
Supplement: Supplementary file 1 [file pathogens-09-00195-s001.pdf]

Fig. S1 Phylogenetic relationships as determined by MLST data for the 123 *C. freundii* isolates from this study and two previous studies [10, 16]. Cluster divisions are marked and were consistent with our previous division [16]. Numbers on or near the nodes are bootstrap values from 1,000 replicates and shown if ≥50%. The tree was constructed using neighbor joining algorithm. ST indicate sequence types.

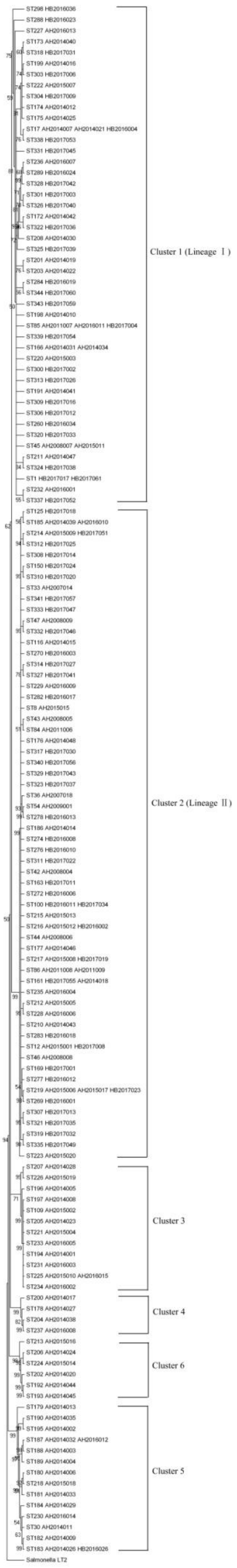
ST298 HB2016036
ST288 HB2016023
ST227 AH2016013
ST173 AH2014040
ST318 HB2017031
ST199 AH2014016
ST303 HB2017006
ST222 AH2015007
ST304 HB2017009
ST174 AH2014012
ST175 AH2014025
ST17 AH2014007 AH2014021 HB2016004
ST338 HB2017053
ST331 HB2017045
ST236 AH2016007
ST289 HB2016024
ST328 HB2017042
ST301 HB2017003
ST326 HB2017040
ST172 AH2014042
ST322 HB2017036
ST208 AH2014030
ST325 HB2017039
ST201 AH2014019
ST203 AH2014022
ST284 HB2016019
ST344 HB2017060
ST343 HB2017059
ST198 AH2014010
ST85 AH2011007 AH2016011 HB2017004
ST339 HB2017054
ST166 AH2014031 AH2014034
ST220 AH2015003
ST300 HB2017002
ST313 HB2017026
ST191 AH2014041
ST309 HB2017016
ST306 HB2017012
ST260 HB2016034
ST320 HB2017033
ST45 AH2008007 AH2015011
ST211 AH2014047
ST324 HB2017038
ST1 HB2017017 HB2017061
ST232 AH2016001
ST337 HB2017052
Cluster 1 (Lineage Ⅰ)
ST125 HB2017018
ST185 AH2014039 AH2016010
ST214 AH2015009 HB2017051
ST312 HB2017025
ST308 HB2017014
ST150 HB2017024
ST310 HB2017020
ST33 AH2007014
ST341 HB2017057
ST333 HB2017047
ST47 AH2008009
ST332 HB2017046
ST116 AH2014015
ST270 HB2016003
ST314 HB2017027
ST327 HB2017041
ST229 AH2016009
ST282 HB2016017
ST8 AH2015015
ST43 AH2008005
ST84 AH2011006
ST176 AH2014048
ST317 HB2017030
ST340 HB2017056
ST329 HB2017043
ST323 HB2017037
ST36 AH2007018
ST54 AH2009001
ST278 HB2016013
ST186 AH2014014
ST274 HB2016008
ST276 HB2016010
ST311 HB2017022
ST42 AH2008004
ST163 HB2017011
ST272 HB2016006
ST100 HB2016011 HB2017034
ST215 AH2015013
ST216 AH2015012 HB2016002
ST44 AH2008006
ST177 AH2014046
ST217 AH2015008 HB2017019
ST86 AH2011008 AH2011009
ST161 HB2017055 AH2014018
ST235 AH2016004
ST212 AH2015005
ST228 AH2016006
ST210 AH2014043
ST283 HB2016018
ST12 AH2015001 HB2017008
ST46 AH2008008
ST169 HB2017001
ST277 HB2016012
ST219 AH2015006 AH2015017 HB2017023
ST269 HB2016001
ST307 HB2017013
ST321 HB2017035
ST319 HB2017032
ST335 HB2017049
ST223 AH2015020
Cluster 2 (Lineage Ⅱ)
ST207 AH2014028
ST226 AH2015019
ST196 AH2014005
ST197 AH2014008
ST109 AH2015002
ST205 AH2014023
ST221 AH2015004
ST233 AH2016005
ST194 AH2014001
ST231 AH2016003
ST225 AH2015010 AH2016015
ST234 AH2016002
Cluster 3
ST200 AH2014017
ST178 AH2014027
ST204 AH2014038
ST237 AH2016008
Cluster 4
ST213 AH2015016
ST206 AH2014024
ST224 AH2015014
ST202 AH2014020
ST192 AH2014044
ST193 AH2014045
Cluster 6
ST179 AH2014013
ST190 AH2014035
ST195 AH2014002
ST187 AH2014032 AH2016012
ST188 AH2014003
ST189 AH2014004
ST180 AH2014006
ST218 AH2015018
ST181 AH2014033
ST184 AH2014029
ST230 AH2016014
ST30 AH2014011
ST182 AH2014009
ST183 AH2014026 HB2016026
Cluster 5
Salmonella LT2

Table S1 List of clonal complexes of *Citrobacter* isolates from this study and the public MLST database[#]

CCs	STs	Isolates	species	Source	Year	Country/location
1	288	HB2016023	*C. freundii*	food	2016	Hebei Province
1	17	AH2014007	*C. freundii*	diarrheal patient	2014	Anhui Province
1	17	AH2014021	*C. freundii*	diarrheal patient	2014	Anhui Province
1	17	HB2016004	*C. freundii*	diarrheal patient	2016	Hebei Province
1	17	NMI_5868/12	*C. freundii*	skin necrosis	2012	Poland
1	17	NMI_5220/12	*C. freundii*	urine	2012	Poland
1	17	M818	*C. freundii*	rectal swab	2008	Latvia
1	17	M829	*C. freundii*	rectal swab	2009	Latvia
2	170	2017523	*C. freundii*	wound	2017	Spain
2	220	AH2015003	*C. freundii*	diarrheal patient	2015	Anhui Province
3	168	N17-00150	*C. freundii*	water	2015	Canada
3	304	HB2017009	*C. freundii*	diarrheal patient	2017	Hebei Province
4	416	ME170049C	*C. freundii*	Rectal	2017	Ireland
4	213	AH2015016	*C. freundii*	diarrheal patient	2015	Anhui Province
5	289	HB2016024	*C. freundii*	food	2016	Hebei Province
5	328	HB2017042	*C. freundii*	food	2017	Hebei Province
5	236	AH2016007	*C. freundii*	diarrheal patient	2016	Anhui Province
6	344	HB2017060	*C. freundii*	food	2017	Hebei Province
6	331	HB2017045	*C. freundii*	food	2017	Hebei Province
7	1	HB2017017	*C. freundii*	diarrheal patient	2017	Hebei Province
7	1	HB2017061	*C. freundii*	food	2017	Hebei Province
7	1	CF65	*C. freundii*	goat	2011	Henan Province
7	1	CF89	*C. freundii*	human	2011	Henan Province
7	1	CF90	*C. freundii*	diarrheal patient	2011	Henan Province
7	1	CF86	*C. freundii*	diarrheal patient	2011	Henan Province
7	1	CF86	*C. freundii*	diarrheal patient	2011	Henan Province
7	1	CF80	*C. freundii*	diarrheal patient	2011	Henan Province
7	1	CF81	*C. freundii*	diarrheal patient	2011	Henan Province
7	1	CF77	*C. freundii*	chicken	2011	Henan Province
7	1	CF73	*C. freundii*	fly	2011	Henan Province
7	1	CF69	*C. freundii*	chopping board	2011	Henan Province
7	1	CF75	*C. freundii*	swine	2011	Henan Province
7	3	CF76	*C. freundii*	fly	2011	Henan Province
7	3	CF79	*C. freundii*	diarrheal patient	2011	Henan Province
7	3	CF93	*C. freundii*	diarrheal patient	2011	Henan Province
7	3	CF92	*C. freundii*	diarrheal patient	2011	Henan Province
8	214	AH2015009	*C. freundii*	diarrheal patient	2015	Anhui Province
8	214	HB2017051	*C. freundii*	food	2017	Hebei Province
8	321	HB2017035	*C. freundii*	environment	2017	Hebei Province
9	332	HB2017046	*C. freundii*	food	2017	Hebei Province
9	265	/	*C. freundii*	/	/	Brazil

9	69	/	*C. freundii*	/	/	Beijing
10	192	AH2014044	*C. freundii*	human	2014	Anhui Province
10	193	AH2014045	*C. freundii*	human	2014	Anhui Province
11	176	AH2014048	*C. freundii*	human	2014	Anhui Province
11	139	/	*C. freundii*	/	/	Beijing
11	6	CF74	*C. freundii*	goat	2011	Henan Province
12	270	HB2016003	*C. freundii*	diarrheal patient	2016	Hebei Province
12	167	N15-02052	*C. freundii*	water	2015	Canada
13	310	HB2017020	*C. freundii*	diarrheal patient	2017	Hebei Province
13	150	HB2017024	*C. freundii*	diarrheal patient	2017	Hebei Province
13	150	/	*C. freundii*	/	/	Canada
13	14	M2308	*C. freundii*	rectal swab	2010	Israel
13	14	IMR208/13	*C. freundii*	urine	2013	Malaysia
13	14	IMR511/13	*C. freundii*	tissue	2013	Malaysia
13	14	IMR503/13	*C. freundii*	tissue	2013	Malaysia
14	341	HB2017057	*C. freundii*	food	2017	Hebei Province
14	494	/	*C. freundii*	/	/	France
15	276	HB2016010	*C. freundii*	environment	2016	Hebei Province
15	261	/	*C. freundii*	/	/	Sevilla
16	216	AH2015012	*C. freundii*	diarrheal patient	2015	Anhui Province
16	216	HB2016002	*C. freundii*	diarrheal patient	2016	Hebei Province
16	274	HB2016008	*C. freundii*	food	2016	Hebei Province
16	403	/	*C. freundii*	/	/	Shandong Province
16	118	Cit fre 80	*C. freundii*	rectal swab	2016	Italy
16	404	/	*C. freundii*	/	/	Singapore
16	119	Cit fre 145	*C. freundii*	rectal swab	2016	Italy
17	212	AH2015005	*C. freundii*	diarrheal patient	2015	Anhui Province
17	228	AH2016006	*C. freundii*	diarrheal patient	2016	Anhui Province
18	44	AH2008006	*C. freundii*	food	2008	Anhui Province
18	415	ME160401H	*C. freundii*	toe	2016	Ireland
19	187	AH2016012	*C. freundii*	diarrheal patient	2016	Anhui Province
19	189	AH2014004	*C. freundii*	diarrheal patient	2014	Anhui Province
19	243	CB00004	*C. freundii*	urine	2017	USA
20	182	AH2014009	*C. freundii*	diarrheal patient	2014	Anhui Province
20	183	AH2014026	*C. freundii*	diarrheal patient	2014	Anhui Province
21	42	AH2008004	*C. freundii*	food	2008	Anhui Province
21	18	M8128	*C. freundii*	rectal swab	2010	Greece
21	18	CF_14	*C. freundii*	urine	2014	Spain
21	18	CF-2316	*C. freundii*	bronchial aspirate	2016	Poland
21	159	Ci32	*C. freundii*	cat	2016	Japan
22	36	AH2007018	*C. freundii*	diarrheal patient	2007	Anhui Province
22	54	AH2009001	*C. freundii*	food	2009	Anhui Province
22	278	HB2016013	*C. freundii*	food	2016	Hebei Province
23	163	HB2017011	*C. freundii*	diarrheal patient	2017	Hebei Province

23	163	N16-02392	*C. freundii*	water	2015	Canada
23	406	/	*C. freundii*	/	/	Shandong Province
24	46	AH2008008	*C. freundii*	food	2008	Anhui Province
24	144	CPO20150025	*C. freundii*	urine	2015	Denmark
24	481	29001247	*C. freundii*	unknown	2019	The Netherlands
24	395	/	*C. freundii*	/	/	Korea
24	508	/	*C. freundii*	/	/	Germany
24	519	/	*C. freundii*	/	/	France
25	269	HB2016001	*C. freundii*	diarrheal patient	2016	Hebei Province
25	219	AH2015006	*C. freundii*	diarrheal patient	2015	Anhui Province
25	219	AH2015017	*C. freundii*	human	2015	Anhui Province
25	219	HB2017023	*C. freundii*	diarrheal patient	2017	Hebei Province
26	283	HB2016018	*C. freundii*	food	2016	Hebei Province
26	62	/	*C. freundii*	/	/	Republic of South Africa
26	91	CF_7	*C. freundii*	urine	2012	Spain
26	91	C-M5-U-060615	*C. freundii*	urine	2015	USA
27	86	AH2011008	*C. freundii*	food	2011	Anhui Province
27	86	AH2011009	*C. freundii*	food	2011	Anhui Province
27	124	Cf5976/16	*C. freundii*	catheter	2016	Poland
28	73	AH2009013	*C. youngae*	diarrheal patient	2009	Anhui Province
28	74	AH2009014	*C. youngae*	diarrheal patient	2009	Anhui Province
28	74	HB2017021	*C. youngae*	diarrheal patient	2017	Hebei Province
28	76	AH2009016	*C. youngae*	food	2009	Anhui Province
29	38	AH2007021	*C. youngae*	diarrheal patient	2007	Anhui Province
29	39	AH2007022	*C. youngae*	diarrheal patient	2007	Anhui Province
29	39	AH2007024	*C. youngae*	food	2007	Anhui Province
29	39	AH2007025	*C. youngae*	food	2007	Anhui Province
29	39	AH2008001	*C. youngae*	food	2008	Anhui Province
29	39	AH2008002	*C. youngae*	food	2008	Anhui Province
29	40	AH2007023	*C. youngae*	human	2007	Anhui Province
30	48	AH2008010	*C. youngae*	food	2008	Anhui Province
30	187	HB2016028	*C. youngae*	diarrheal patient	2016	Hebei Province
31	60	AH2009008	*C. youngae*	food	2009	Anhui Province
31	71	AH2009010	*C. youngae*	food	2009	Anhui Province
31	72	AH2009011	*C. youngae*	food	2009	Anhui Province
32	75	AH2009015	*C. youngae*	diarrheal patient	2009	Anhui Province
32	77	AH2009017	*C. youngae*	food	2009	Anhui Province
33	51	AH2008014	*C. braakii*	food	2008	Anhui Province
33	55	AH2009002	*C. braakii*	food	2009	Anhui Province
34	58	AH2009006	*C. braakii*	food	2009	Anhui Province
34	225	HB2017095	*C. braakii*	food	2017	Hebei Province
35	79	AH2010001	*C. braakii*	food	2010	Anhui Province
35	385	HB2017108	*C. braakii*	food	2017	Hebei Province

36	297	HB2016035	*C. braakii*	food	2016	Hebei Province
36	297	HB2017099	*C. braakii*	environment	2017	Hebei Province
36	355	HB2017072	*C. braakii*	diarrheal patient	2017	Hebei Province
37	353	HB2017070	*C. braakii*	diarrheal patient	2017	Hebei Province
37	370	HB2017090	*C. braakii*	food	2017	Hebei Province
38	369	HB2017089	*C. braakii*	food	2017	Hebei Province
38	378	HB2017101	*C. braakii*	food	2017	Hebei Province
39	381	HB2017104	*C. braakii*	food	2017	Hebei Province
39	384	HB2017107	*C. braakii*	food	2017	Hebei Province

[#] Clonal complexes were defined as STs which shared six of the seven gene alleles. We also extracted all isolates and STs of *C. fruendii* from the public MLST database. There are no *C. youngae* or *C. braakii* isolates/STs available from the MLST database except the deposition from our current and previous studies. Clonal complexes were numbered from 1 to 39. ST: sequence types; CCs: clonal complexes; /: no data.

Table S2 Prevalence of MDR isolates in different *Citrobacter* species

Species	MDR isolates/all isolates (%)				
	Diarrheal patients	Foods	Healthy individuals	Environment	Total
C. freundii	28/94 (29.8%)	22/41 (63.7%)	7/19 (36.8%)	1/7 (14.3%)	58/161 (36.0)
C. youngae	5/30 (16.7%)	6/24 (25.0%)	1/3 (33.3%)	0	12/57 (21.1)
C.braakii	1/8 (12.5%)	22/40 (55.0%)	0/1 (0)	3/4 (75.0%)	26/53 (49.1)

Table S3 Prevalence of Adherence and Cytotoxicity in 128 *Citrobacter* Isolates

Adhesion	Cytotoxicity, Number (%)			Total Number (%)
	High	Intermediate	None	
High	4 (3.1)	7 (5.5)	3 (2.3)	14 (10.9)
Intermediate	7 (5.5)	23 (18.0)	27 (21.1)	57 (44.5)
Low	2 (1.6)	9 (7.0)	26 (20.3)	37 (28.9)
None	0 (0)	1 (0.8)	19 (14.8)	20 (15.6)
All	13 (10.2)	40 (31.3)	75 (58.6)	128 (100)

Table S4 Source, drugs resistance, Genotypes and Anibiotic resistance phenotype of 128 *Citrobacter* Isolates

Isolates	Year	Source	STs	NDR	Antibiotic resistance phenotype
HB2016001	2016	Diarrheal patient	269	1	(CFX)
HB2016002	2016	Diarrheal patient	216	3	(AMP)(CTX,CAZ,CFX,TIO)(AZM)
HB2016003	2016	Diarrheal patient	270	1	(CFX)
HB2016006	2016	Food packing slipway	272	1	(CFX)
HB2016008	2016	Chicken	274	8	（AMP）(CTX,FEP,CFX,TIO)(NAL)(GEN,STR)(TET)(CHL)(Sul,SXT)(AMZ)
HB2016010	2016	Filling staircase	276	1	(CFX)
HB2016011	2016	Pork	100	1	(CFX)
HB2016012	2016	Chicken	277	2	（AMP）(CFX)
HB2016013	2016	Duck	278	3	（AMP)(CFX) (MEM)
HB2016017	2016	Duck	282	1	(NAL)
HB2016018	2016	Chicken	283	2	（AMP）(CFX)
HB2017001	2017	Diarrheal patient	169	2	(CFX)(CT)
HB2017008	2017	Diarrheal patient	12	5	(AMP)(CTX,CFX)(TET)(CHL)(SXT)
HB2017011	2017	Diarrheal patient	163	7	(AMP)(CTX,CAZ,CFX)(AZM)(NAL,CIP,LEV)(STR)(TET,DOX)(CHL)(SUL,SXT)
HB2017013	2017	Diarrheal patient	307	1	(CFX)
HB2017014	2017	Diarrheal patient	308	1	(CFX)
HB2017018	2017	Diarrheal patient	125	2	(CFX)(NAL)
HB2017019	2017	Diarrheal patient	217	1	(CFX)
HB2017020	2017	Diarrheal patient	310	1	(CFX)
HB2017022	2017	Diarrheal patient	311	2	(AMP)(CFX)
HB2017023	2017	Diarrheal patient	219	3	(AMP)(CTX,CAZ,CFX,TIO)(AZM)
HB2017024	2017	Diarrheal patient	150	1	(CFX)
HB2017025	2017	Diarrheal patient	312	3	(AMP)(TET)(CHL)
HB2017027	2017	Diarrheal patient	314	4	(AMP)(CFX)(NAL)(TET)
HB2017030	2017	Diarrheal patient	317	1	(TIO)
HB2017032	2017	Diarrheal patient	319	5	(AMP)(CFX)(TET)(CHL)(SUL,SXT)
HB2017034	2017	Feeder sole	100	1	(CFX)
HB2017035	2017	Feeder floor	321	1	(CFX)
HB2017037	2017	Feeder floor	323	1	(CFX)
HB2017041	2017	Cucumber	327	1	(CFX)
HB2017043	2017	Minced pork	329	1	(CFX)
HB2017046	2017	Minced pork	332	2	（AMP）(CFX)
HB2017047	2017	Minced pork	333	6	（AMP)(CFX,CTX)(GEN,STR)(TET)(CHL)(SUL,SXT)
HB2017049	2017	Minced pork	335	2	（AMP）(CFX)
HB2017051	2017	Minced pork	214	2	(CFX)(TET)
HB2017055	2017	Minced pork	161	6	（AMP)(CFX)(NAL)(TET)(SXT)(AMZ)
HB2017056	2017	Minced pork	340	4	（AMP)(CFX)(NAL)(TET,DOX)
HB2017057	2017	Minced pork	341	4	(CFX)(TET)(CHL)(SUL,SXT)
HB2016004	2016	Diarrheal patient	17	2	(AMP)(CFX)
HB2016019	2016	Pork	284	9	(AMP)(CTX,FEP,CFX,TIO)(MEN)(NAL,LEV)(STR)(TET,DOX)(CHL)(SUL,SXT)(AMZ)
HB2016023	2016	Pork	288	9	(AMP)(CTX,CAZ,FEP,CFX,TIO)(AZM)(NAL,CIP,LEV)(GEN,KAN,STR)(TET,DOX)(CHL)(SUL,SXT)(AMZ)
HB2016024	2016	Vegetables	289	3	(AMP)(CTX,CFX,TIO)(NAL,CIP,LEV)

HB2016034	2016	Chicken	260	7	(AMP)(CTX,CFX,TIO)(NAL)(STR)(TET,DOX)(CHL)(SUL,SXT)
HB2016036	2016	Diarrheal patient	298	2	(AMP)(CTX,CFX)
HB2017002	2017	Diarrheal patient	300	1	(CFX)
HB2017003	2017	Diarrheal patient	301	2	(AMP)(CFX)
HB2017004	2017	Diarrheal patient	85	2	(AMP)(CFX)
HB2017006	2017	Diarrheal patient	303	2	(CFX)(STR)
HB2017009	2017	Diarrheal patient	304	4	(AMP)(CTX,CFX)(TET)(CHL)
HB2017012	2017	Diarrheal patient	306	1	(CFX)
HB2017016	2017	Diarrheal patient	309	1	(CFX)
HB2017017	2017	Diarrheal patient	1	2	(AMP)(CFX)
HB2017026	2017	Diarrheal patient	313	6	(AMP)(TET)(NAL,CIP)(TET,DOX)(CHL)(SUL,SXT)
HB2017031	2017	Diarrheal patient	318	2	(CFX)(AMZ)
HB2017033	2017	Feeder floor	320	0	
HB2017036	2017	Feeder floor	322	3	(AMP)(CFX)(STR)
HB2017038	2017	Minced pork	324	4	(CFX)(KAN)(TET,DOX)(SUL,SXT)
HB2017039	2017	Minced pork	325	2	（AMP）(CFX)
HB2017040	2017	Leek	326	8	(AMP)(CTX,CFX,TIO)(NAL)(STR)(TET,DOX)(CHL)(SUL,SXT)(AMZ)
HB2017042	2017	Pork	328	9	(AMP)(CTX,CAZ,FEP,CFX,TIO)(IMI,MEN)(NAL,CIP,LEV)(GEN,STR)(TET,DOX)(CHL)(SUL,SXT)(AMZ)
HB2017045	2017	Pork	331	7	(AMP)(CTX,CFX,TIO)(NAL)(GEN,STR)(TET)(CHL)(SUL,SXT)
HB2017052	2017	Salad	337	2	（AMP）(CFX)
HB2017053	2017	Pork	338	4	(AMP)(CTX,CAZ,CFX,TIO)(AZM)(STR)
HB2017054	2017	Cold noodle	339	2	(AMP)(CFX)
HB2017059	2017	Minced pork	343	7	(AMP)(CTX,FEP,CFX,TIO)(AMZ)(GEN,KAN,STR)(TET)(CHL)(SUL,SXT)
HB2017060	2017	Pork	344	7	(AMP)(CTX,FEP,CFX,TIO)(NAL)(GEN,STR)(TET,DOX)(CHL)(SUL,SXT)
HB2017061	2017	Minced pork	1	2	(AMP)(CFX)
HB2016015	2016	Chicken	280	5	(CFX)(NAL)(TET)(CHL)(SUL,SXT)
HB2016032	2016	Feeder sole	295	5	(AMP)（CTX,CFX)(STR)(CHL)(SUL,SXT)
HB2016033	2016	Duck	296	2	(CFX)(NAL)
HB2016035	2016	Chicken	297	6	(AMP)(CTX,CFX,TIO)(NAL,CIP,LEV)(TET,DOX)(CHL)(SUL,SXT)
HB2017044	2017	Minced pork	330	2	（AMP）(CFX)
HB2017048	2017	Minced pork	334	2	(CFX)(TET,DOX)
HB2017062	2017	Minced pork	345	2	(AMP)(CFX)
HB2017068	2017	Minced pork	351	2	(AMP)(CFX)
HB2017070	2017	Diarrheal patient	353	2	(CFX)(NAL)
HB2017071	2017	Diarrheal patient	354	2	(AMP)(CFX)
HB2017072	2017	Diarrheal patient	355	1	(CFX)
HB2017074	2017	Diarrheal patient	356	2	(AMP)(CFX)
HB2017072	2017	Diarrheal patient	357	1	(CFX)
HB2017076	2017	Diarrheal patient	358	4	(AMP)(CFX)(NAL)(TET,DOX)
HB2017077	2017	Diarrheal patient	357	1	(CFX)
HB2017078	2017	Diarrheal patient	359	1	(NAL,CIP)
HB2017079	2017	Feeder floor	360	3	(AMP)(CFX)(NAL)
HB2017081	2017	Feeder floor	362	2	(AMP)(CFX)
HB2017082	2017	Chicken	363	1	(STR)

HB2017083	2017	Pork	364	1	(CHL)
HB2017084	2017	Pork	365	3	(CFX)(NAL)(TET)
HB2017087	2017	Pork	367	8	(AMP)(CTX,CFX,TIO)(AZM)(NAL)(STR)(TET,DOX)(CHL)(SUL,SXT)
HB2017089	2017	Minced pork	369	2	(CFX)(SXT)
HB2017090	2017	Pork	370	8	(AMP)(CTX,CFX,TIO)(AZM))(NAL,CIP,LEV)(GEM,STR)(TET,DOX)(SUL,SXT)(AMZ)
HB2017091	2017	Minced pork	371	3	（CFX)(NAL)(TET,DOX)
HB2017092	2017	Minced pork	372	4	(AMP)(CFX)(NAL)(STR)
HB2017093	2017	Minced pork	373	2	（CFX)(TET,DOX)
HB2017094	2017	Minced pork	81	3	(CFX)(IMI)(AMZ)
HB2017095	2017	Minced pork	225	6	(AMP)(CTX,CFX,RIO)(NAL)(TET,DOX)(CHL)(SUL,SXT)
HB2017096	2017	Pork	374	6	(AMP)(CTX,CAZ,FEP,CFX,TIO)(AZM)(STR)(TET,DOX)(SUL,SXT)
HB2017097	2017	Salad	375	0	
HB2017098	2017	Pork	376	1	(CFX)
HB2017099	2017	Floor	297	6	(AMP)（CTX,FEP,CFX,TIO)(NAL,CIP,LEV)(STR)(DOX)(SUL,SXT)
HB2017100	2017	Minced pork	377	2	(CFX)(STR)
HB2017101	2017	Pork	378	2	(CFX)(TET)
HB2017102	2017	Minced pork	379	3	(AMP)(CFX)(NAL)
HB2017103	2017	Minced pork	380	4	(AMP)(CFX)(NAL)(TET,DOX)
HB2017104	2017	Minced pork	381	3	(AMP)(CFX)(TET)
HB2017105	2017	Pork	382	9	(AMP)(CTX,CFX,TIO)(AZM)(NAL,CIP,LEV)(GEM,STR)(TET)(CHL)(SUL,SXT)(AMZ)
HB2017106	2017	Minced pork	383	4	(CFX)(NAL)(TET,DOX)（SXT）
HB2017107	2017	Minced pork	384	7	(AMP)(CFX)(NAL)(KAN,STR)(TET,DOX)(CHL)(SUL,SXT)
HB2017108	2017	Minced pork	385	6	(AMP)(CTX,FEP,CFX,TIO)(AZM)(NAL)(TET,DOX)(SUL,SXT)
HB2017109	2017	Buckwheat cake	386	1	(CFX)
HB2017110	2017	Salad	375	0	
HB2017111	2017	Green pepper	387	2	(AMP)(CFX)
HB2016029	2016	Diarrheal patient	292	1	(CFX)
HB2017007	2017	Diarrheal patient	237	8	(AMP)(CTX,CAZ,CFX)(AZM)(NAL,CIP,LEV)(KAN,STR)(TET,DOX)(CHL)(SUL,SXT)
HB2017021	2017	Diarrheal patient	74	3	(AMP)(TET)(SUL,SXT)
HB2017029	2017	Diarrheal patient	316	2	(AMP)(CFX)
HB2017067	2017	Diarrheal patient	350	1	(CFX)
HB2017086	2017	Minced pork	258	2	(AMP)(CFX)
HB2016026	2016	Diarrheal patient	183	1	(CFX)
HB2016027	2016	Diarrheal patient	291	1	(CFX)
HB2016028	2016	Diarrheal patient	187	0	
HB2016031	2016	Duck	294	2	(AMP) (CFX)
HB2017063	2017	Diarrheal patient	346	2	(AMP)(CFX)
HB2017064	2017	Diarrheal patient	347	0	
HB2017065	2017	Diarrheal patient	348	7	(AMP)(CFX)(NAL)(STR)(TET,DOX)(CHL)(SUL)
HB2017066	2017	Diarrheal patient	349	0	
HB2017069	2017	Egg cake	352	2	(AMP)(SUL)
HB2017085	2017	Minced pork	366	1	(CFX)

ST: sequence types; NDR: number of drugs resistant to.
